# *Bacillus licheniformis*YB06: A Rhizosphere–Genome-Wide Analysis and Plant Growth-Promoting Analysis of a Plant Growth-Promoting Rhizobacterium Isolated from *Codonopsis pilosula*

**DOI:** 10.3390/microorganisms12091861

**Published:** 2024-09-08

**Authors:** Shuo Ni, Yamiao Wu, Ning Zhu, Feifan Leng, Yonggang Wang

**Affiliations:** School of Life Science and Engineering, Lanzhou University of Technology, Lanzhou 730050, China; adszsthl@outlook.com (S.N.); niskeyanshunli@gmail.com (Y.W.); 221081700010@lut.edu.cn (N.Z.); lff0928@sina.com (F.L.)

**Keywords:** plant growth-promoting rhizobacteria, *Bacillus licheniformis*, genome-wide analysis, biofertilizer, rhizosphere soil bacterial community

## Abstract

*Codonopsis pilosula*, commonly known as Dangshen, is a valuable medicinal plant, but its slow growth and susceptibility to environmental stress pose challenges for its cultivation. In pursuit of sustainable agricultural practices to enhance the yield and quality of Dangshen, the present study isolated a bacterial strain exhibiting plant growth-promoting potential from the rhizosphere of *C. pilosula*. This strain was subsequently identified as *Bacillus licheniformis*YB06. Assessment of its plant growth-promoting attributes revealed the potential of *B. licheniformis* YB06 as a biofertilizer. Whole-genome sequencing of *B. licheniformis* YB06 revealed a genome size of 4,226,888 bp with a GC content of 46.22%, harboring 4325 predicted protein-coding sequences. Genomic analysis of *B. licheniformis* YB06 revealed a diverse array of genes linked to induced systemic resistance (ISR) and plant growth-promoting (PGP) traits, encompassing phytohormone production, nitrogen assimilation and reduction, siderophore biosynthesis, phosphate solubilization, biofilm formation, synthesis of PGP-related amino acids, and flagellar motility. Seed germination assays demonstrated the positive effects of *B. licheniformis* YB06 on the germination and growth of *C. pilosula* seedlings. Furthermore, we explored various fertilization regimes, particularly the *B. licheniformis* YB06-based biofertilizer, were investigated for their impact on the structure and diversity of the *C. pilosula* rhizosphere soil bacterial community. Our findings revealed that fertilization significantly impacted soil bacterial composition and diversity, with the combined application of *B. licheniformis* YB06-based biofertilizer and organic fertilizer exhibiting a particularly pronounced enhancement of rhizosphere bacterial community structure and diversity. This study represents the first report on the beneficial effects of *B. licheniformis* YB06 on both the growth of *C. pilosula* and the composition of its rhizosphere soil microbial community. These findings provide a theoretical foundation and practical guidance for the development of novel bio-organic compound fertilizers, thereby contributing to the sustainable cultivation of *C. pilosula*.

## 1. Introduction

*Codonopsis pilosula* (commonly known as Dangshen) is a perennial herbaceous species within the *Campanulaceae* family. It has a long history of cultivation in East Asia, where its roots are utilized for both culinary and medicinal purposes [1,2]. The significant medicinal value of *C. pilosula* in traditional Chinese medicine, coupled with its antibacterial, anti-inflammatory, and anti-cancer properties revealed by modern scientific research, has spurred growing interest in enhancing its yield and expanding its cultivation [3,4]. Currently, economical and inexpensive chemical pesticides and fertilizers are widely used to increase crop productivity and control plant diseases [5]. However, the excessive application of chemical fertilizers and pesticides can lead to a decline in the concentration of bioactive compounds in medicinal plants like *C. pilosula*. Furthermore, it can contribute to the development of pesticide resistance in pathogenic microorganisms, resulting in plant diseases and a cascade of agricultural and environmental safety concerns [6,7]. The use of plant growth-promoting rhizobacteria (PGPR) to enhance soil fertility, suppress plant diseases, and promote agricultural plant growth is a promising sustainable alternative environmental strategy [8,9,10,11,12]. Furthermore, the combined application of biofertilizer (utilizing PGPR as the primary raw material) and organic fertilizer presents a more economically viable, rapidly effective, and sustainable approach to enhance both the yield of medicinal plants and the concentration of their bioactive compounds [13,14]. Numerous bacterial species have been utilized globally as alternatives to conventional fertilizers and chemical pesticides [15,16]. However, reports on the use of PGPR and corresponding biofertilizers to enhance the cultivation of *C. pilosula* remain scarce. 

Previous research has demonstrated that certain members of the isolates of the genus *Bacillus* possess the capacity to function as potent biocontrol agents and PGPR [17,18,19]. *Bacillus amyloliquefaciens* GB03 has been demonstrated to stimulate growth and enhance abiotic stress tolerance in Arabidopsis thaliana [20]. *Bacillus subtilis* QST713 is extensively employed as a biocontrol agent in agricultural practices, notably in the cultivation of *Agaricus bisporus*. Han et al. [21] demonstrated that *Bacillus velezensis* FZB42 exhibits significant antagonistic activity against *Phytophthora sojae*, the causal agent of soybean Phytophthora root rot (PRR). While numerous studies have substantiated the efficacy of various *Bacillus* species in promoting plant growth and disease suppression, research exploring their application in medicinal plants, particularly in the cultivation of *C. pilosula*, and their impact on the rhizosphere microbiome remains limited.

To identify plant growth-promoting rhizobacteria (PGPR) suitable for *C. pilosula* cultivation, a promising strain was selected from our lab’s previous collection of bacteria isolated from the *C. pilosula* rhizosphere soil. This strain was identified as *Bacillus licheniformis*and designated as *B. licheniformis YB06*. The *B. licheniformis* YB06 strain was isolated from a specific ecological niche (the rhizosphere of *C. pilosula*), and its unique plant growth-promoting properties and genomic characteristics remain unexplored. The similarities and differences in functions and genes between this strain and known *B. licheniformis* strains have yet to be elucidated. The present study aims to address this knowledge gap by comprehensively investigating the plant growth-promoting potential, whole-genome sequence, and impact on the rhizosphere bacterial community of this novel *B. licheniformis* strain isolated from the rhizosphere of *C. pilosula*. Following the selection and identification of the target strain, the growth-promoting capabilities of *B. licheniformis* YB06 were systematically assessed under various conditions. Whole-genome sequencing was conducted, and a comprehensive analysis of growth-promoting genes and metabolic pathways was performed. Our analysis revealed that *B. licheniformis* YB06 possesses plant growth-promoting (PGP) potential, and subsequent experiments demonstrated its ability to stimulate seed germination and enhance seedling growth in *C. pilosula*. Furthermore, we explored the combined effects of a biofertilizer containing *B. licheniformis* YB06 as the main active ingredient and various fertilization regimes on the rhizosphere bacterial community structure and diversity in *C. pilosula*. Our findings indicate that the combined application of organic and biofertilizers significantly influenced soil physicochemical properties and bacterial diversity. This study represents the beneficial effects of *B. licheniformis* YB06 on *C. pilosula* seed germination, seedling growth, and the composition of its rhizosphere soil microbial community. These findings provide a theoretical foundation and practical guidance for the development of novel bio-organic compound fertilizers, thereby contributing to the sustainable cultivation of *C. pilosula*.

## 2. Materials and Methods

### 2.1. Species Identification and Biological Characteristics Determination of B. licheniformis YB06

#### 2.1.1. Strain Material

*Bacillus licheniformis*YB06 was isolated from the rhizosphere soil of healthy *Codonopsis pilosula* plants (sourced from Weiyuan County, Gansu Province, China) grown in a laboratory soil culture room during previous studies (single colonies were isolated via serial dilution plating and subsequently purified through streak plating). The strain is currently preserved in the Laboratory of Microbiology and Synthetic Biology, Lanzhou University of Technology. The *C. pilosula* seeds used in this study were collected from Weiyuan County, Gansu Province, China. The seeds had been stored for less than one year prior to the experiment.

#### 2.1.2. Morphological and Molecular Biological Identification of *B. licheniformis* YB06

Single colonies of *B. licheniformis* YB06 were obtained by streak plating on nutrient agar. Colony morphology was assessed based on size, shape, and pigmentation. Gram staining was performed using a BKMAMLAB Gram Staining Kit from Changde Bkman Biotechnology Co., Ltd. (Changde, China). Bacterial genomic DNA was extracted using the Solarbio Bacterial Genomic DNA Extraction Kit from Solarbio Science & Technology Co., Ltd. (Beijing, China). The 16S rRNA gene was amplified by PCR using universal primers 27F and 1492R [22], and the resulting amplicons were sequenced by Guangzhou Gene Denovo Biotechnology Co., Ltd. (Ijamsville, MD, USA). The obtained 16S rRNA gene sequences were compared with those in the NCBI GenBank database (http://www.ncbi.nlm.nih.gov, accessed on 15 January 2024) using BLASTn for taxonomic identification.

#### 2.1.3. Determination of Biological Characteristics of *B. licheniformis* YB06

*B. licheniformis* YB06 was revived on LB agar plates at 37 °C for 24 h. Activated culture was inoculated (1% *v*/*v*) into LB broth with varying pH (4–10) and NaCl (0–7%) concentrations and incubated at 37 °C, 180 rpm for 24 h. Growth was assessed at different temperatures (10–37 °C) using a spectrophotometer. IAA production was quantified using the Salkowski assay, with a standard curve of 0.5–25.0 mg·L^−1^ IAA [23]. Siderophore production was assessed using the CAS assay [24]. Nitrogen fixation was tested on nitrogen-free JNFb agar plate. The strain cultures were incubated at 37 °C, 180 rpm for 3–5 days. The nitrogen content was confirmed by a Kjeldahl analyzer. ACC deaminase activity was assessed using the ninhydrin assay [25]. Hydrolytic enzyme production (cellulase, xylanase, protease, and amylase) was tested on agar plates containing specific substrates by inoculating *B. licheniformis* YB06 and incubating at 37 °C for 5 days [26,27,28]. For antibiotic susceptibility, *B. licheniformis* YB06 was grown to mid-log phase, spread on LB agar, and tested using disc diffusion with various antibiotics, including rifampicin, kanamycin, chloramphenicol, ampicillin, tetracycline, and spectinomycin hydrochloride. The D/d ratio was calculated to assess resistance. The D/d ratio is calculated by dividing the diameter of the zone of inhibition (D) by the diameter of the antibiotic disc (d). A smaller D/d ratio indicates a higher level of resistance, while a larger D/d ratio indicates greater sensitivity.

#### 2.1.4. Determination of Plant Growth Promotion (PGP) of *B. licheniformis* YB06 with *C. pilosula* Seeds

A single colony of *B. licheniformis* YB06 was inoculated into 100 mL LB broth and incubated overnight at 37 °C with shaking at 180 rpm. The culture was harvested at the early logarithmic phase (OD_600_ = 0.6) by centrifugation at 8000 r/min for 10 min. The cell pellet was washed three times with sterile water and resuspended in sterile water. The bacterial cell suspension was then serially diluted to obtain OD_600_ values of 0.25, 0.5, 0.75, 1.0, and 1.25. A bacterial cell density of 1.0 × 10^8^ colony-forming units per milliliter (CFU/mL) corresponded to an OD_600_ of 1.0. Based on this correlation, cultures exhibiting OD_600_ values of 0.25, 0.5, 0.75, and 1.25 were estimated to contain 0.25 × 10^8^, 0.5 × 10^8^, 0.75 × 10^8^, and 1.25 × 10^8^ CFU/mL, respectively. *C. pilosula* seeds were surface-sterilized with 2% sodium hypochlorite (NaClO) and 75% ethanol. The sterilized seeds were then divided into six groups: five treatment groups immersed in *B. licheniformis* YB06 suspensions at various OD_600_ values (0.25, 0.5, 0.75, 1.0, and 1.25) for 12 h and a control group immersed in sterile water for 12 h. The treated seeds were subsequently plated on Petri dishes lined with filter paper moistened with sterile water and a layer of cotton (50 seeds per Petri dish, a density determined to be optimal for germination and seedling growth in preliminary experiments). The Petri dishes were then sealed with parafilm to minimize moisture loss and incubated in a growth chamber maintained at a relative humidity of 50% for 14 days. The moisture level in the Petri dishes was checked daily, and sterile water was added as needed to ensure the filter paper and cotton remained consistently moist.

### 2.2. Comprehensive Whole-Genome Sequencing of B. licheniformis YB06

#### 2.2.1. Preparation, Sequencing, and Assembly of Genomic DNA

Genomic DNA was extracted from *B. licheniformis* YB06 using the HiPure Bacterial DNA Kit (Guangzhou, China). DNA quality was assessed using a Qubit fluorometer and a NanoDrop spectrophotometer. PacBio long-read and Illumina short-read sequencing were performed by Guangzhou Gene Denovo Biotechnology Co., Ltd. Continuous long reads generated by SMRT sequencing were de novo assembled using the Falcon assembler. Raw reads from the Illumina platform were subjected to quality control using FASTP (V 0.20.0) with the following parameters: (1) removal of reads containing ≥10% ambiguous bases (N); (2) removal of reads with ≥50% bases having a Phred quality score ≤ 20; and (3) removal of reads aligning to barcode adapters. The resulting high-quality reads were used to polish the genome assembly generated by Falcon, and the final genome sequence was determined using Pilon.

#### 2.2.2. Gene Prediction, Functional Annotation, and Analysis of Plant Growth-Promoting Information

Gene prediction was performed using the NCBI Prokaryotic Genome Annotation Pipeline (PGAP). Non-coding RNAs, including rRNAs, tRNAs, and small RNAs (sRNAs), were identified using rRNAmmer, tRNAscan-SE, and Cmsearch, respectively. Genomic islands were predicted using IslandPath-DIMOB. CRISPR-Cas systems were identified using CRISPRCasFinder. Putative prophage regions were detected using PHASTER (PHAge Search Tool Enhanced Release). Gene annotation was performed by aligning predicted protein sequences against the NCBI non-redundant (Nr), Swiss-Prot, KEGG, Gene Ontology (GO), and Clusters of Orthologous Groups (COG) databases. Additional functional annotation was conducted using the Carbohydrate-Active enZYmes Database (CAZy), Virulence Factor Database (VFDB), and the Comprehensive Antibiotic Resistance Database (CARD). Gene Ontology (GO) terms were assigned to predicted proteins using Blast2GO software (version: 6.0.0). The genome of *B. licheniformis* YB06 was further compared with the GO and eggNOG databases to identify and characterize genes potentially involved in plant growth promotion.

### 2.3. Analysis of Rhizosphere Bacterial Diversity of C. pilosula under Different Fertilization Treatments

#### 2.3.1. Experimental Fertilizers and Soil Substrates

The ternary compound fertilizer, with a nitrogen (N), phosphorus (P), and potassium (K) ratio of 15:12:10, was supplied by Lanzhou Liuquan Forestry Compound Fertilizer Factory. Organic fertilizer, primarily composed of composted cow manure (N + P_2_O_5_ + K_2_O ≥ 5.0%, organic matter ≥ 50%, pH 5.5–8.5, moisture ≥ 30%), was sourced from Gansu Runyi Biotechnology Co., Ltd. The compost was not sterilized before application to simulate field conditions. The biofertilizer, in granular form, containing a consortium of *B. subtilis* and *B. licheniformis* (viable count ≥ 1.0 × 10^9^ CFU/g, moisture < 10%), was produced by Gansu Shangnong Biotechnology Co., Ltd. The soil used for the pot experiments was collected from a field used for *C. pilosula* cultivation (consistent with the seed source, Gansu Province, China). The basic nutrient levels of the pot experiment soil were as follows: total nitrogen approximately 2.0 g/kg, total phosphorus 0.3 g/kg, total potassium 25 g/kg, available phosphorus 12 mg/kg, available nitrogen 0.13 g/kg, available potassium 0.14 g/kg, and organic matter 1.8 mg/kg.

#### 2.3.2. Determination of Soil Physical and Chemical Properties under Different Fertilization Regimes

Four fertilization treatment groups were established: (1) chemical fertilizer (CF) group: 0.1% compound fertilizer, soil to vermiculite ratio of 4:1, and 0.2 g of urea; (2) chemical and organic fertilizer (CFOF) group: soil, organic fertilizer, and vermiculite in a ratio of 3:1:1, supplemented with 0.2 g of urea and 0.05% compound fertilizer; (3) nitrogen fertilizer (NF) group: soil to vermiculite ratio of 4:1, with 0.2 g of urea; (4) organic fertilizer and biofertilizer (OFBOF) group: soil, organic fertilizer, and vermiculite in a ratio of 3:1:1, supplemented with 0.2 g of urea and 1.0 g of biofertilizer containing *B. licheniformis* YB06 Each treatment group consisted of three 1 kg pots, with each pot containing approximately 1 g of *C. pilosula* seeds (ensuring equal seed numbers across pots), the respective soil/amendment mixtures as described, and 55% moisture. The experiment included three replicates per treatment group. Soil samples were collected after 30 days (three replicates per group). Soil moisture was determined gravimetrically. For EC, pH, and salinity, 4 g of soil was mixed with 20 mL of deionized water, shaken, and settled. Supernatant pH and EC were measured, with salinity calculated from EC. Soil organic matter (SOM), total nitrogen (TN), total phosphorus (TP), and total potassium (TK) were analyzed after air-drying and sieving. SOM was determined by the potassium dichromate method [29], TN by the Kjeldahl method [30], TP by the molybdenum-antimony colorimetric method [31], and TK by flame photometry [32]. Available nitrogen (AN), available phosphorus (AP), and available potassium (AK) were quantified using atomic absorption spectrophotometry [33]. Soil genomic DNA was extracted using the E.Z.N.A.^®^ Soil DNA Kit, quality was assessed by agarose gel electrophoresis, and concentration/purity was determined using a NanoDrop 2000 spectrophotometer (Thermo Fisher Scientific, Waltham, MA, USA) [34].

#### 2.3.3. Extraction of Soil Bacterial DNA and Illumina MiSeq Sequencing

Soil genomic DNA was extracted using the E.Z.N.A.^®^ Soil DNA Kit (Omega Bio-tek, Norcross, GA, USA). DNA quality was assessed by agarose gel electrophoresis (1%), and DNA concentration and purity were determined using a NanoDrop 2000 spectrophotometer (Thermo Fisher Scientific, Waltham, MA, USA) [34]. Paired-end sequencing was performed on the Illumina MiSeq platform (Illumina, San Diego, CA, USA) following the manufacturer’s instructions. Sequencing services were provided by Guangzhou Gene Denovo Biotechnology Co., Ltd.

#### 2.3.4. Data Analysis of Soil Bacterial Diversity

Statistical analyses were performed using SPSS 19.0 software (IBM Corp., Armonk, NY, USA). A one-way analysis of variance (ANOVA) was used to assess significant differences among treatments (*p* < 0.05). Alpha diversity indices were visualized using GraphPad Prism 9 (GraphPad Software, San Diego, CA, USA). Beta diversity was assessed by principal coordinate analysis (PCoA) and hierarchical clustering, and redundancy analysis (RDA) was performed to identify relationships between bacterial community composition and environmental variables. These analyses were conducted using the BioLadder online platform (https://www.bioladder.cn, accessed on 10 May 2024). Linear discriminant analysis effect size (LEfSe) was employed to identify differentially abundant bacterial taxa among treatments using the Galaxy-based LEfSe online tool (http://huttenhower.sph.harvard.edu.cn, accessed on 20 May 2024).

## 3. Results

### 3.1. Identification and Biological Characteristics of B. licheniformis YB06

#### 3.1.1. Morphological and Molecular Characterization of *B. licheniformis* YB06

**Figure 1:**
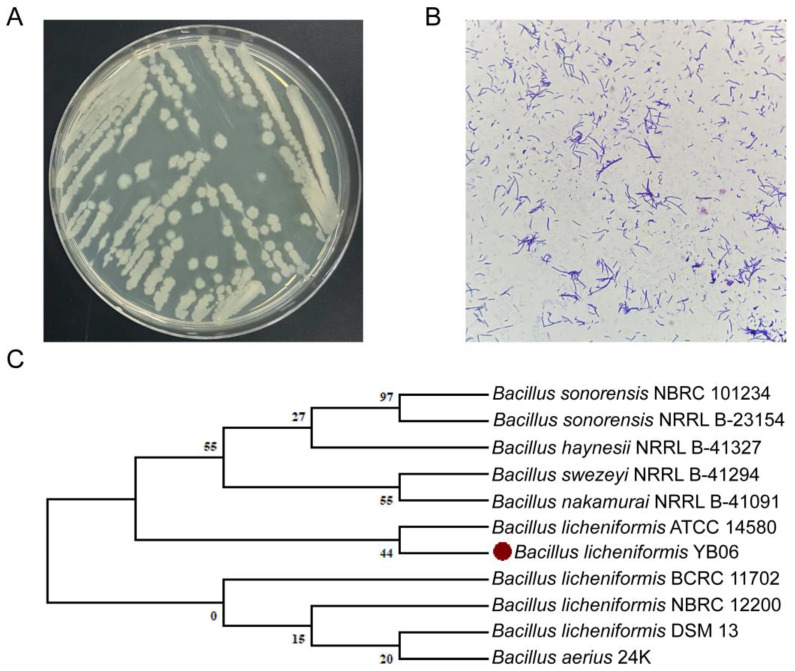
(**A**) Colony morphology of *B. licheniformis* YB06 on LB agar, (**B**) Gram staining of *B. licheniformis* YB06 cells visualized under a light microscope (10 × 40 magnification), (**C**) Phylogenetic tree based on 16S rRNA gene sequences, showing the relationship of *B. licheniformis* YB06 (marked with a red dot) with other closely related *Bacillus* spp.

As shown in Figure 1A, the strain exhibited robust growth on LB agar, forming light yellow, non-glossy colonies. Gram staining revealed that the cells were Gram-positive rods (Figure 1B). Phylogenetic analysis based on the 16S rRNA gene sequence confirmed the taxonomic placement of the strain within the *Bacillus licheniformis*clade. Phylogenetic analysis based on 16S rRNA gene sequences resolved all *Bacillus licheniformis*strains included in the tree into two distinct clades. Our strain of interest, *B. licheniformis* YB06, clustered closely with the type strain *B. licheniformis* ATCC 14580 in one clade, while the remaining *B. licheniformis* strains formed a separate clade with *Bacillus aerius* 24K (Figure 1C). Based on the morphological and molecular characterization, the strain was identified as *Bacillus licheniformis*and designated as *B. licheniformis* YB06.

#### 3.1.2. Determination of the Biological Characteristics of *B. licheniformis* YB06

**Figure 2:**
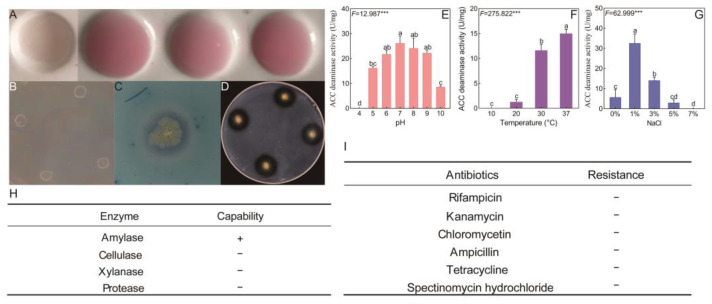
Assessment of the biological characteristics of *B. licheniformis* YB06: (**A**) Qualitative detection of IAA production by *B. licheniformis* YB06; (**B**) Nitrogen fixation capacity of *B. licheniformis* YB06; (**C**) Siderophore production by *B. licheniformis* YB06; (**D**) Amylase activity of the *B. licheniformis* YB06; (**E**) ACC deaminase ability of *B. licheniformis* YB06 under varying pH conditions; (**F**) ACC deaminase ability of *B. licheniformis* YB06 under varying temperature conditions; (**G**) ACC deaminase ability of *B. licheniformis* YB06 under varying NaCl concentrations; (**H**) Extracellular enzyme production capacity; (**I**) antibiotic resistance evaluation. In (**E**–**G**), different lowercase letters above the bars denote statistically significant differences (*p* < 0.05) between treatments as determined by Tukey’s test. Additionally, *** above the figures signifies a highly significant difference, with *p* < 0.001.

*B. licheniformis* YB06 growth was significantly affected by pH (*p* < 0.01), with optimal growth at pH 7–8 and no growth at pH 4 (Figure S1A). Growth increased with temperature, peaking at 37 °C (Figure S1B). Optimal growth occurred at 1% NaCl, with inhibition at concentrations exceeding 11% (Figure S1C). IAA production varied significantly under different conditions (*p* < 0.001), peaking at pH 7, 30 °C, and 1% NaCl (Figure 2A and Figure S2A–C). *B. licheniformis* YB06 exhibited nitrogen fixation ability (Figure 2B), with efficiency influenced by pH, temperature, and NaCl concentration (*p* < 0.001), peaking at pH 7, 37 °C, and 1% NaCl (Figure S2D–F). Siderophore production was observed (Figure 2C), with optimal production at pH 8 and increasing with temperature (Figure S2G,H). Siderophore production was maintained even at high NaCl concentrations (Figure S2I). ACC deaminase production was significantly influenced by environmental conditions (*p* < 0.01), peaking at pH 7 and increasing with temperature, and was highest at 1% NaCl (Figure 2E–G). *B. licheniformis* YB06 exhibited amylase activity (Figure 2D,H) and was sensitive to all tested antibiotics (Figure 2I).

#### 3.1.3. Effect of the *B. licheniformis* YB06 on the Growth of *C. pilosula* Seedlings

Figure S3A illustrates the effect of different concentrations of *B. licheniformis* YB06 on *C. pilosula* seed germination. A morphological analysis revealed that *B. licheniformis* YB06 treatment influenced root length and thickness in *C. pilosula* seedlings. The addition of *B. licheniformis* YB06 did not significantly affect the final germination percentage of *C. pilosula* seeds (Figure S3B). However, at CFU/mL values below 1.0 × 10^8^, *B. licheniformis* YB06 significantly enhanced the germination potential by 28.75%, 17.54%, and 17.39%, respectively, compared to the control (Figure S3C). The highest germination potential (53.33%) was observed at 0.25 × 10^8^ CFU/mL. While *B. licheniformis* YB06 significantly enhanced germination potential at lower concentrations (CFU/mL < 1.0 × 10^8^), this positive effect diminished at higher concentrations (CFU/mL > 1.0 × 10^8^), suggesting a potential inhibitory effect at higher bacterial densities. Figure S3D illustrates the significant effect (*p* < 0.05) of different *B. licheniformis* YB06 concentrations on *C. pilosula* seedling root length. At 0.25 × 10^8^ CFU/mL and 0.5 × 10^8^ CFU/mL, root length increased by 15.91% and 30.19%, respectively, compared to the control. However, it is important to note that at CFU/mL values above 0.5 × 10^8^ CFU/mL, *B. licheniformis* YB06 treatment exerted a negative impact, leading to a decrease in root length. The fresh weight of *C. pilosula* seedlings was also significantly affected by *B. licheniformis* YB06 treatment, with increases of 8.66%, 19.91%, 12.52%, 15.30%, and 7.91% observed at different CFU/mL values compared to the control (Figure S3E). The highest fresh weight (2.3 mg) was observed at 5.0 × 10^7^ CFU/mL.

### 3.2. Whole-Genome Analysis of B. licheniformis YB06 

#### 3.2.1. Genomic Features and Comparative Genomic Analysis of *B. licheniformis* YB06

The complete genome of *B. licheniformis* YB06 was 4,226,888 base pairs (bp) in size, with a G + C content of 46.22%. The genome contained 4160 predicted genes, including 4024 protein-coding sequences, 24 rRNA genes organized into three operons (16S-23S-5S), and 81 tRNA genes.

**Figure 3:**
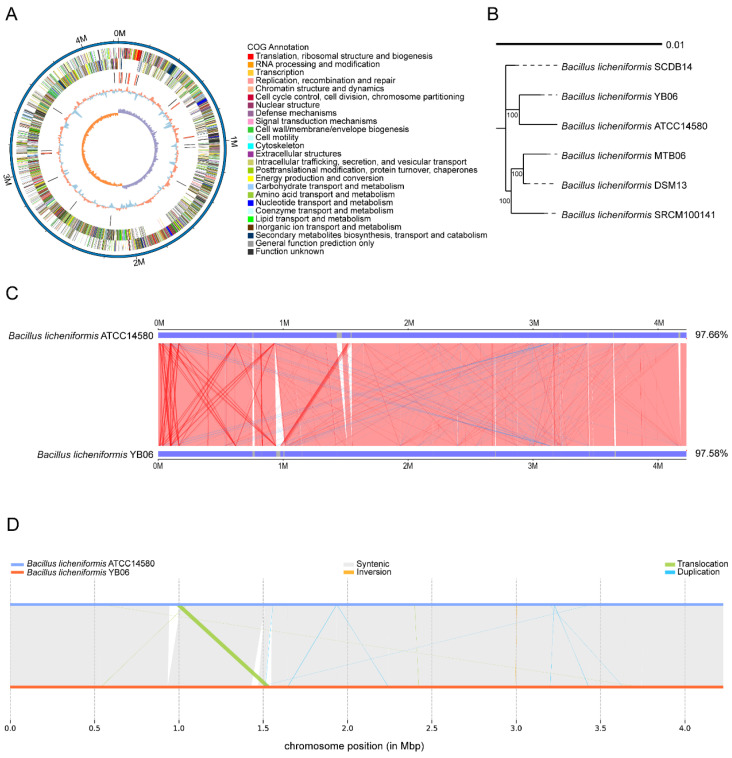
(**A**) Circular map of the *B. licheniformis* YB06 genome; (**B**) Phylogenetic tree based on the whole genome of *B. licheniformis* YB06; (**C**) Synteny analysis between *B. licheniformis* YB06 and *B. licheniformis* ATCC14580; (**D**) Structural variation (SV) comparison between *B. licheniformis* YB06 and *B. licheniformis* ATCC14580.

Figure 3A shows the circular genome map of *B. licheniformis* YB06. The genes were classified into 25 COG categories, with a majority associated with functions such as amino acid transport metabolism, transcription, inorganic ion transport and metabolism, and the biosynthesis, transport, and catabolism of secondary metabolites. The *B. licheniformis* YB06 genome was predicted to contain 24 rRNA genes, 81 tRNA genes, and 31 sRNA genes (Table S1). The rRNA genes were organized into three operons, each containing one copy of the 16S, 23S, and 5S rRNA genes. The average lengths of the 16S, 23S, and 5S rRNA genes were 1550 bp, 2930 bp, and 116 bp, respectively, representing 0.29%, 0.55%, and 0.02% of the total genome length. The average lengths of the tRNA and sRNA genes were 77 bp and 101 bp, respectively, accounting for 0.15% and 0.07% of the genome. A total of six genomic islands (GIs), designated GI1 to GI6, were predicted within the *B. licheniformis* YB06 genome (Table S2). The total length of these GIs was 158,735 bp, with an average length of 26,455.83 bp. The number of genes and their putative functions within each GI are summarized in Table S3. Specifically, GI_1 primarily encodes proteins involved in nucleotide metabolism and DNA recombination. GI_2 appears to be associated with phage infection. GI_3 mainly encodes proteins involved in signal transduction and regulation. GI_4 contains genes related to amino acid metabolism and redox reactions. GI_5 harbors genes encoding proteases involved in protein degradation and turnover. GI_6 primarily encodes proteins related to nucleic acid and small-molecule metabolism. The presence of these diverse GIs underscores the importance of horizontal gene transfer in shaping the genome of this bacterium, contributing to its adaptability and evolutionary success. The prediction of GIs provides a theoretical and data foundation for further research to verify the functions of these GIs and their encoded proteins. This will contribute to elucidating their specific roles in bacterial physiology, virulence, and environmental interactions. Additionally, three prophage regions (Prophage_001 to Prophage_003), totaling 127,367 bp in length, were predicted to be latent prophages in the genome (Table S3). No CRISPR-Cas systems were detected in the genome of *B. licheniformis* YB06.

Phylogenetic analysis based on the whole-genome sequence of *B. licheniformis* YB06 placed it in the same clade as the type strain *B. licheniformis* ATCC 14580 (Figure 3B). Comparative genomic analysis revealed a high degree of synteny between *B. licheniformis* YB06 and *B. licheniformis* ATCC 14580, with a similarity of 97.58% and a coverage rate of 97.66% (Figure 3C). The analysis identified 18 large-scale syntenic blocks (SYN) and 48 small-scale syntenic blocks (SYNAL) shared between the two genomes. A structural variation (SV) analysis identified seven duplications, four translocations, and one inversion in the genome of *B. licheniformis* YB06 compared to the type strain *B. licheniformis* ATCC 14580 (Figure 3D). These genomic rearrangements may contribute to phenotypic and functional differences between the two strains.

#### 3.2.2. Functional Genomic Annotation of *B. licheniformis* YB06 Genome

**Figure 4:**
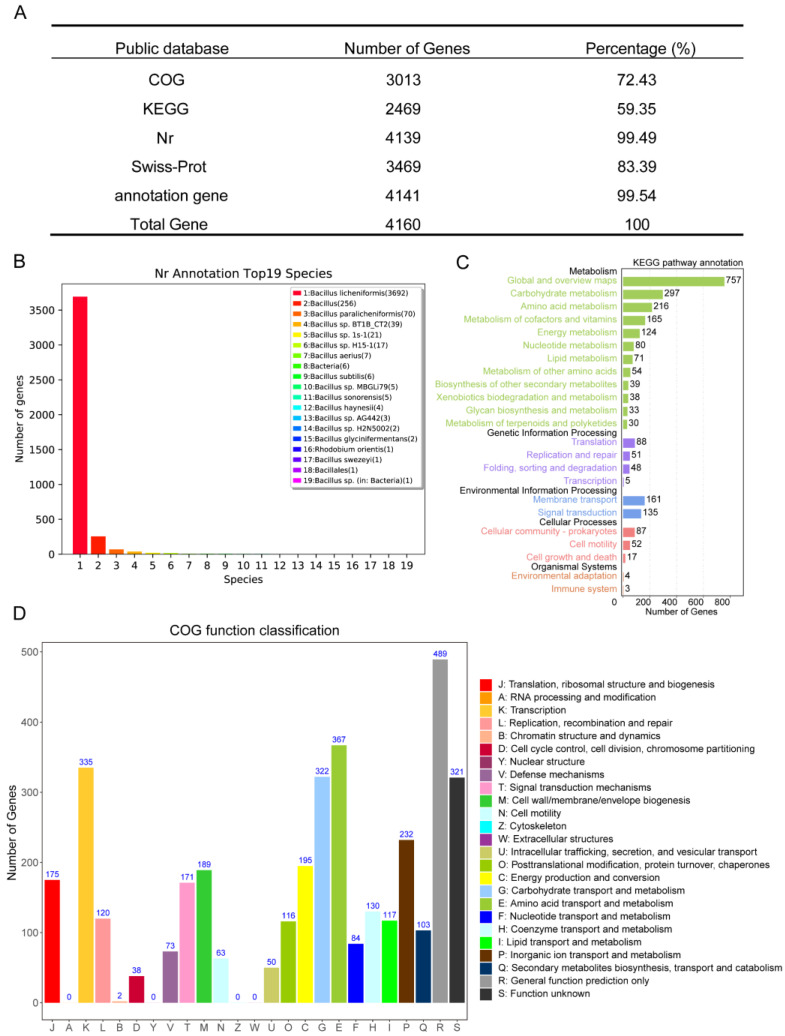
(**A**) Summary of genome annotation results; (**B**) Species distribution annotated in the Nr database; (**C**) Bar chart of whole-genome KEGG annotation classifications; (**D**) Bar chart of whole-genome COG annotations for *B. licheniformis* YB06.

A total of 2246 genes were annotated against the Nr, Swiss-Prot, Clusters of Orthologous Groups (COG), and KEGG databases, representing 99.54% of all annotated genes (Figure 4A). The Nr annotation (Figure 4B) showed that most *B. licheniformis* YB06 genes (3692) were similar to *Bacillus licheniformis*, with 256 genes similar to other *Bacillus* spp., confirming *B. licheniformis* YB06 as *Bacillus licheniformis*. The KEGG annotation of the *B. licheniformis* YB06 genome identified genes in five major functional classes: metabolism, genetic information processing, environmental information processing, cellular processes, and organismal systems (Figure 4C). The 20 most enriched KEGG pathways (Figure S4A) included metabolism, biosynthesis, flagellar assembly, transporters, and two-component systems. This suggests a diverse metabolic capacity, including the ability to utilize a variety of carbon sources and synthesize essential biomolecules. The presence of genes associated with flagellar assembly and various transporters indicates the potential for motility and active nutrient uptake, contributing to its adaptation to diverse environments. Notably, the identification of genes related to nitrogen metabolism aligns with the observed nitrogen fixation capability of *B. licheniformis* YB06, highlighting its potential role in promoting plant growth and development. 

The Gene Ontology (GO) analysis revealed that the majority of the annotated genes in *B. licheniformis* YB06 were associated with biological processes, followed by cellular components and molecular functions (Figure S4B). Figure 4D shows the Clusters of Orthologous Groups (COG) functional classification of the *B. licheniformis* YB06 genome. A significant proportion of genes are associated with fundamental cellular processes, including amino acid transport and metabolism, transcription, and carbohydrate transport and metabolism. Additionally, a substantial number of genes are involved in inorganic ion transport and metabolism, energy production and conversion, and cell wall/membrane biogenesis. COG analysis revealed a predominance of genes encoding enzymes involved in amino acid biosynthesis and catabolism, which participate in nutrient metabolism, secondary metabolite production, and phytohormone biosynthesis. Notably, the high abundance of COG4915 (5-bromo-4-chloroindolyl phosphate hydrolase), an enzyme implicated in indole-3-acetic acid (IAA) biosynthesis, suggests that *B. licheniformis YB06* may promote plant growth through the production of this phytohormone and other growth-promoting substances. Carbohydrate-active enzymes (CAZymes) are a diverse group of enzymes involved in the synthesis, modification, and degradation of carbohydrates. Numerous bacteria utilize CAZymes to break down plant biomass and generate various growth-promoting compounds. The genome of *B. licheniformis* YB06 encodes a repertoire of CAZymes, belonging to six distinct CAZyme families (Figure S5A). The presence of these enzymes suggests that *B. licheniformis* YB06 has the potential to colonize plant tissues, elicit plant defense mechanisms, and protect against microbial pathogens. As illustrated in Figure S5B, the genome predicts 10 gene clusters associated with the biosynthesis of secondary metabolites. These secondary metabolite clusters, linked to lipopeptide antibiotics, possess the potential to inhibit the growth of pathogenic fungi. 

Based on annotation results from the Virulence Factor Database (VFDB), we identified five genes associated with stress response and protein quality control in the *B. licheniformis* YB06 genome: *clpC*, *clpB*, *htpB*, *clpP*, and *invA* (Table S4). These genes encode proteins involved in various stress responses and protein quality control, suggesting that *B. licheniformis* YB06 may utilize these mechanisms to antagonize plant pathogens. Annotation of the *B. licheniformis* YB06 genome using the Comprehensive Antibiotic Resistance Database (CARD) revealed the presence of several genes conferring resistance to various antibiotic classes, including fluoroquinolones, cephalosporins, rifamycins, macrolides, and tetracyclines. Additionally, multiple genes encoding multidrug efflux pumps belonging to the Major Facilitator Superfamily (MFS), Resistance-Nodulation-Division (RND), and ATP-binding cassette (ABC) families were identified (Table S5). The presence of these antibiotic resistance genes is consistent with the intrinsic resistance commonly observed in rhizosphere microorganisms. Their presence may contribute to the intrinsic resistance of *B. licheniformis* YB06 and could potentially be upregulated under microbial stress conditions, thus likely contributing to its survival and competitive advantage in the rhizosphere.

#### 3.2.3. Metabolic Pathway Analysis of Plant Growth Promotion in *B. licheniformis* YB06

**Figure 5:**
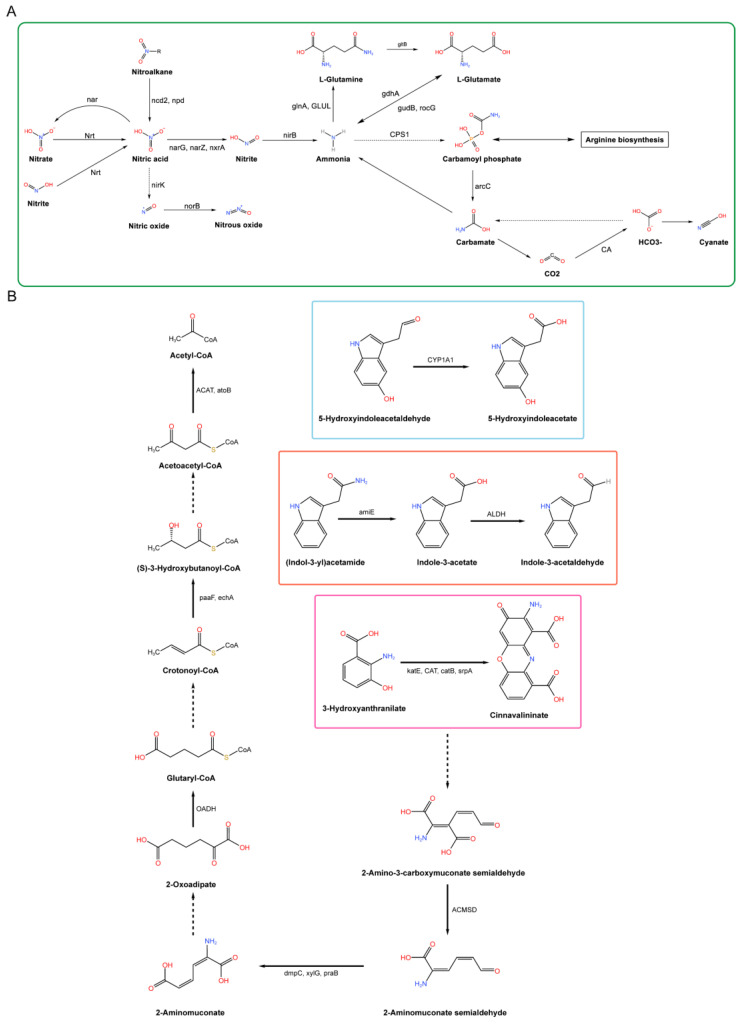
(**A**) Nitrogen metabolism pathway in *B. licheniformis* YB06. (**B**) Tryptophan metabolism pathway in *B. licheniformis* YB06.

As shown in Table 1, *B. licheniformis* YB06 encompasses various metabolic pathways and corresponding key genes associated with plant growth promotion. Figure S6 illustrates the KEGG annotation results of the carbon metabolism pathway in *B. licheniformis* YB06, identifying key enzymes involved in glycolysis and the synthesis of organic acids, such as pyruvate kinase (*pyk*) and acetate kinase (*ackA*). The KEGG nitrogen metabolism annotation results of the *B. licheniformis* YB06 genome are shown in Figure 5A. Enzymes associated with nitrogen metabolism, such as carbamate kinase (*arcC*), were annotated. The genome of *B. licheniformis* YB06 also contains genes encoding nitrate reductase and nitrite reductase. The presence of genes encoding nitrate reductase, nitrite reductase, and related transport proteins suggests that *B. licheniformis* YB06 can reduce nitrate to nitrite and subsequently reduce nitrite to ammonium, which can be assimilated by plants. Figure 5B depicts the tryptophan metabolism pathway in *B. licheniformis* YB06. The whole genome of *B. licheniformis* YB06 contains genes related to IAA synthesis, including formaldehyde dehydrogenase (*ALDH*), amidase (*amiE*), and others. Amidase and formaldehyde dehydrogenase are key enzymes in the indole-3-acetamide and indole-3-acetaldehyde pathways, respectively, catalyzing the hydrolysis of indole-3-acetamide and indole-3-acetaldehyde to indole-3-acetic acid. The pathway reveals that *B. licheniformis* YB06 can synthesize tryptophan, histidine, tryptophan, serine, L-cysteine, methionine, threonine, glutamine, arginine, ornithine, lysine, valine, leucine, and isoleucine, with all necessary enzymes encoded in its genome (Figure S7). Figure S8 shows that *B. licheniformis* YB06 carries genes encoding siderophore synthesis, including key genes *entA*, *entB*, and *entC*. *B. licheniformis* YB06 only contains the *MbtH* gene for side-chain synthesis. Our analysis identified motility-related genes in *B. licheniformis* YB06. The genome includes genes for flagellar biosynthesis proteins such as *flhA*, flagellar hook genes *flgK* and *flgL*, and critical chaperone proteins for flagellar assembly, such as *fliT*. Additionally, it contains flagellar proteins *flhA*, *fliD*, and *fliI*, which include the flagellar M-ring protein *FliF*, flagellar cap protein *FliD*, flagellar biosynthesis proteins *FlhA* and *FlhB*, flagellar motility proteins *MotA* and *MotB*, and cluster motility proteins (Figure S9). Additionally, the genome revealed the presence of chemotaxis-related proteins and genes (Figure S10), including methyl-accepting chemotaxis proteins (*MCPs*), chemotaxis protein methyltransferase (*CheR*), and sensor kinase (*CheA*), among others. The collective presence of these enzymes and proteins is likely to significantly enhance host-plant perception and recognition, facilitating efficient root colonization and the establishment of a conducive microenvironment, enabling *B. licheniformis* YB06 to successfully colonize the plant rhizosphere.

### 3.3. Physicochemical Characteristics of Soil under Various Fertilization Regimes

Our results revealed significant differences in soil physicochemical properties among the different fertilization treatments (Table 2). Specifically, pH, electrical conductivity (EC), and salinity varied significantly (*p* < 0.05) among treatments, although the pH values remained within a relatively narrow range (7.02–7.47). Soil moisture content did not differ significantly among treatments (*p* > 0.05). The application of chemical fertilizer (CF) alone resulted in significantly higher electrical conductivity (EC) and salinity compared to other treatments, reaching maximum values of 377.33 μs/cm and 0.051 mol/L, respectively. Soil total nitrogen (TN) and available nitrogen (AN) content also differed significantly (*p* < 0.05) among treatments. The nitrogen fertilizer (NF) treatment exhibited the highest TN and AN levels, with increases of 45.81%, 26.86%, and 36.58% for TN and 28.13%, 25.35%, and 33.43% for AN compared to the CF, CFOF, and OFBOF treatments, respectively. Soil total phosphorus (TP) was significantly higher in the CF treatment compared to all other treatments (*p* < 0.05). Although not statistically significant (*p* > 0.05), available phosphorus (AP) and total potassium (TK) tended to be higher in the OFBOF treatment. Soil organic matter (SOM) was significantly higher in the CFOF treatment compared to the CF, NF, and OFBOF treatments, with increases of 42.99%, 35.96%, and 10.08%, respectively (*p* < 0.05).

### 3.4. Effects of Different Fertilization Treatments on Rhizosphere Bacterial Diversity of C. pilosula

#### 3.4.1. Analysis of Soil Bacterial Diversity under Different Fertilization Conditions

**Figure 6:**
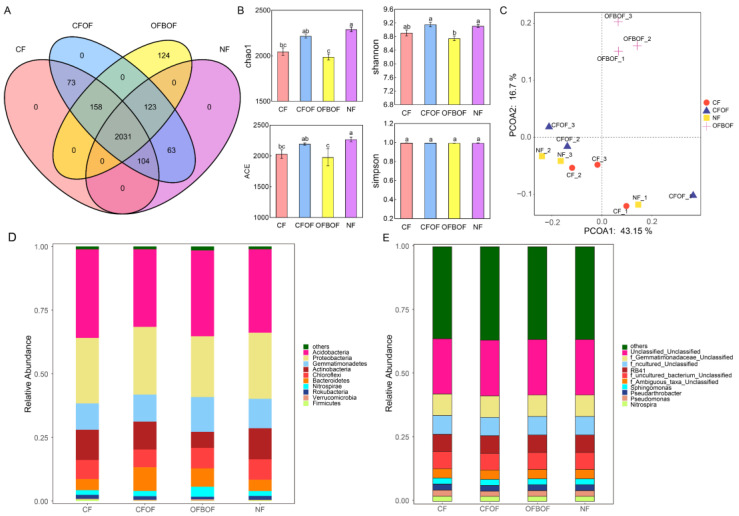
(**A**) Operational taxonomic unit (OTU) clustering of soil bacteria under different fertilization regimes. (**B**) Alpha diversity of rhizosphere soil bacteria associated with *C. pilosula* under different fertilization treatments. (**C**) Principal coordinate analysis (PCoA) based on Bray–Curtis dissimilarity of soil bacterial communities. (**D**) Community composition of soil bacteria at the phylum level under different fertilization regimes. (**E**) Community composition of soil bacteria at the genus level under different fertilization regimes. In (**B**), different lowercase letters above the bars denote statistically significant differences (*p* < 0.05) between treatments as determined by Tukey’s test.

Figure 6A illustrates the results of the operational taxonomic unit (OTU) clustering analysis of soil bacterial communities under different fertilization regimes. A total of 2031 OTUs were identified across all treatments. The OFBOF treatment harbored 124 unique OTUs, while no unique OTUs were found in the other treatments. The highest number of shared OTUs (158) was observed between the OFBOF and CF treatments. The number of observed OTUs at each taxonomic level for each fertilization treatment is presented in Table 3. Fertilization significantly increased the number of OTUs at the phylum, class, order, family, and genus levels, with the highest OTU richness observed in the OFBOF treatment. We then assessed the alpha diversity of the *C. pilosula* rhizosphere bacterial communities under different fertilization regimes (Figure 6B). The Chao1, Shannon, and ACE indices, which reflect microbial richness and diversity, were significantly higher (*p* < 0.05) in the OFCF and NF treatments compared to the CF treatment, with increases of 7.41%, 2.67%, and 7.39% for OFCF and 13.22%, 3.99%, and 12.79% for NF, respectively. Conversely, the OFBOF treatment showed significantly lower (*p* < 0.05) Chao1, Shannon, and ACE indices compared to CF, with decreases of 3.15%, 1.82%, and 2.59%, respectively. The Simpson index, a measure of community evenness, did not differ significantly among treatments. The analysis of β-diversity revealed that fertilization significantly altered the bacterial community structure in the *C. pilosula* rhizosphere. The principal coordinate analysis (PCoA) based on Bray–Curtis dissimilarity (Figure 6C) showed a clear separation of the OFBOF treatment from the CF, CFOF, and NF treatments, with the first two axes explaining 43.15% and 16.7% of the variation, respectively. This indicates that the addition of the biofertilizer to the organic fertilizer (OFBOF) resulted in a distinct shift in the bacterial community composition compared to the other treatments. These findings are consistent with previous studies demonstrating that the application of microbial inoculants can significantly alter soil microbial community structure. Furthermore, our results indicate that the soil microbial community structure exhibits differential responses to microbial inoculants and other fertilizer types.

#### 3.4.2. Soil Bacterial Community Structure under Various Fertilization Regimes

**Figure 7:**
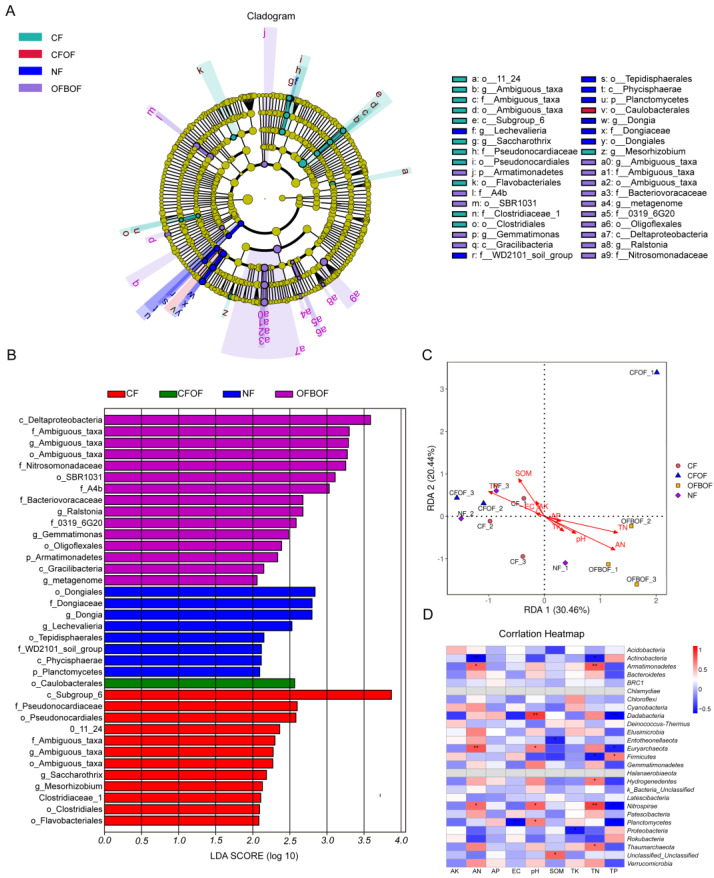
(**A**) Phylogenetic tree illustrating the diversity of soil bacteria. (**B**) Linear discriminant analysis effect size (LEfSe) plot depicting differentially abundant soil bacteria across fertilization treatments. (**C**) Redundancy analysis (RDA) ordination biplot illustrating the relationship between soil bacterial community composition and environmental variables. (**D**) Heatmap representing Spearman rank correlations between abundant soil bacterial taxa and soil physicochemical properties. In (**D**), * indicates a significant difference (*p* < 0.05), ** indicates a more significant difference (*p* < 0.01).

The bacterial community composition at the phylum level was similar across all fertilization treatments (Figure 6D). The dominant phyla were *Acidobacteria*, *Proteobacteria*, *Gemmatimonadetes*, *Actinobacteria*, and *Chloroflexi*, with average relative abundances of 32.94%, 25.55%, 11.55%, 10.34%, and 7.66%, respectively. *Bacteroidetes*, *Nitrospirae*, and *Rokubacteria* were identified as subdominant phyla. Among them, *Acidobacteria* was the most abundant phylum across all treatments, with a consistent relative abundance of approximately 32.95%. *Actinobacteria* exhibited the highest relative abundance (12.19%) in the NF treatment. Compared to the CF treatment, the CFOF treatment resulted in a decrease in the relative abundance of *Acidobacteria* and *Chloroflexi* by 11.67% and 10.10%, respectively and an increase in *Proteobacteria* by 1.60%. The OFBOF treatment led to a decrease in the relative abundance of *Acidobacteria* and *Proteobacteria* by 2.19% and 6.53%, respectively, and a substantial increase in *Gemmatimonadetes* by 34.06%. The NF treatment decreased the relative abundance of *Acidobacteria* by 4.13% and increased that of *Gemmatimonadetes* by 12.56%. As shown in Figure 6E, the relative abundance of soil bacteria at the genus level varied under different microbial fertilizer treatments. An unclassified genus within the Acidobacteria phylum exhibited the highest abundance across all treatments, reaching a maximum of 21.76%. The second most prevalent genus was an unclassified genus belonging to the Gemmatimonadetes phylum, with a relative abundance of 8.44%. To gain deeper insights into the variations in soil bacterial community diversity across different fertilization regimes, linear discriminant analysis effect size (LEfSe) analysis was conducted. The analysis revealed that the primary differentiating taxa were predominantly associated with the organic fertilizer combined with bio-organic fertilizer (OFBOF), no fertilizer (NF), and chemical fertilizer (CF) treatment groups (Figure 7A). Notably, the OFBOF treatment led to a significant enrichment of *Deltaproteobacteria* and *Ambiguous_taxa* (purple), while the NF treatment favored the proliferation of *Dongiales* (blue). The CF treatment resulted in a marked increase in *Caulobacterales* (green), whereas the chemical fertilizer combined with bio-organic fertilizer (CFOF) treatment significantly promoted the abundance of Subgroup_6 (an undefined category group) and *Pseudonocardia* (red) (Figure 7B). 

#### 3.4.3. Correlation between Soil Microbial Community Composition and Environmental Factors

To assess the impact of environmental factors on the soil bacterial community composition within the *C. pilosula* rhizosphere, redundancy analysis (RDA) was employed. The analysis revealed that variations in soil properties induced by different fertilization regimes exerted a significant influence on the structure of the soil bacterial community. In Figure 7C, the initial two axes of the RDA ordination accounted for 30.46% and 20.44% of the total variance in soil bacterial community composition, respectively. Furthermore, Spearman rank correlations were employed to evaluate the relationships between the abundance of dominant bacterial phyla and soil physicochemical properties (Figure 7D). The findings revealed significant negative correlations between *Actinobacteria* abundance and soil available nitrogen (AN) and total nitrogen (TN) content. Conversely, *Armatimonadetes*, *Euryarchaeota*, and *Nitrospirae* exhibited significant positive correlations with soil AN, while *Dadabacteria*, *Euryarchaeota*, *Nitrospirae*, and *Planctomycetes* were positively correlated with both soil TN and total phosphorus (TP). *Firmicutes* abundance was negatively correlated with soil TP (*p* < 0.05). Principal coordinate analysis (PCoA) and RDA results suggested that fertilization regimes may modulate the impact of environmental factors on bacterial community structure, particularly within the rhizosphere. 

## 4. Discussion

### 4.1. Taxonomic Classification and Biological Characteristics of B. licheniformis YB06

In the present study, a plant growth-promoting bacterium isolated from the rhizosphere soil of healthy *C. pilosula* plants was taxonomically characterized using a combination of morphological and molecular approaches. Based on the results, the isolate was identified as *Bacillus licheniformis*and designated as *B. licheniformis* YB06. *Bacillus* spp. are extensively studied as plant endophytic growth-promoting bacteria, renowned for their capacity to synthesize an array of bioactive molecules. These compounds encompass antimicrobial metabolites, siderophores (iron-chelating agents), and various industrially relevant enzymes, including proteases, amylases, alkaline ribonucleases, and penicillinases [35]. Numerous studies have substantiated the plant growth-promoting (PGP) capabilities of *B. licheniformis*. For instance, de O. Nunes et al. [36] reported that *B. licheniformis* can enhance tomato growth, while Bhutani et al. [37] identified a salt-tolerant strain, *B. licheniformis* MHN 12, exhibiting characteristics similar to our *B. licheniformis* YB06 isolate. *B. licheniformis* YB06 demonstrated notable tolerance to salt stress, thriving in media containing up to 1% NaCl. The ability of *B. licheniformis* YB06 to tolerate high salt concentrations may confer an ecological advantage in its natural habitat, enabling it to thrive and promote the growth of *C.pilosula* in high-altitude, saline environments. While salt tolerance is common among rhizosphere microorganisms, this trait also suggests its potential application for promoting the growth of *C. pilosula* or other host crops in new areas with elevated soil salinity, thus expanding the cultivation range of its host plants. Furthermore, Devi et al. [38] demonstrated the ability of *B. licheniformis* to promote rice growth and yield, and Liu et al. [39] documented the positive effects of *B. licheniformis* on potato growth and water use efficiency. Based on these findings, we hypothesize that *B. licheniformis* YB06 may possess analogous growth-promoting and potentially biocontrol properties. Characterization of the biological properties of *B. licheniformis* YB06 revealed that *B. licheniformis* exhibits high stability, with optimal growth occurring at a pH range of neutral to slightly alkaline and a temperature of 37 °C. *C. pilosula* thrives in the arid, high-altitude regions of northwestern China, characterized by intense ultraviolet radiation and significant diurnal temperature fluctuations. Summer midday temperatures can soar above 35 °C, while nighttime temperatures can drop to around 10 °C. The soil in these regions is predominantly neutral to slightly alkaline. The growth characteristics of *B. licheniformis* YB06 indicate its ability to survive in the natural habitat of *C. pilosula* [40,41]. The indole-3-acetic acid (IAA) produced by bacteria not only confers benefits to plants but also indirectly aids bacterial colonization in the rhizosphere by enhancing plant root biomass [42]. Numerous studies have demonstrated the IAA production capacity of *Bacillus* spp. [43,44,45] and their ability to fix atmospheric nitrogen, thereby promoting plant growth. Iron, an essential micronutrient for both plants and microorganisms [46,47,48], is often sequestered by bacterial siderophores, which can directly or indirectly enhance plant growth. Direct mechanisms involve increasing nutrient availability in the soil, while indirect mechanisms include suppressing pathogen growth by limiting iron accessibility [49,50]. Notably, *B. licheniformis* MHN12 has been reported to possess siderophore-producing capabilities [37]. Additionally, 1-aminocyclopropane-1-carboxylate (ACC) deaminase, an enzyme that degrades the ethylene precursor ACC, serves as an indicator of plant growth-promoting rhizobacteria (PGPR) activity. By reducing ethylene levels, ACC deaminase can alleviate plant stress and enhance growth [51,52,53]. Several studies have confirmed the ACC deaminase production capacity of *Bacillus* spp. [54,55,56]. Our investigation revealed that *B. licheniformis* YB06 consistently produced IAA, siderophores, fixed nitrogen, and ACC under all experimental conditions. The maximum values of IAA, siderophore, nitrogen fixation, and ACC deaminase production under varying environmental conditions were 6.9 ± 0.3 μg/mL, 72.8 ± 1.7%, 0.33 ± 0.01%, and 32.6 ± 0.8U/mg, respectively. This indicates that *B. licheniformis* YB06 is a probiotic with growth-promoting potential. Our subsequent germination verification experiments with *C. pilosula* showed that a certain concentration of *B. licheniformis* solution can promote the germination and growth of *C. pilosula* seedlings, significantly promote the germination potential of *C. pilosula*, and also significantly increase the root length of *C. pilosula* seedlings. This confirms that *B. licheniformis* YB06 has the ability to promote seed germination and seedling growth. This is similar to the research results of Medison et al. [57], who found that *B. licheniformis* YZCUO202005 can promote the germination of maize seeds. While our study demonstrates the plant growth-promoting potential of *B. licheniformis* YB06 and provides insights into its genomic basis, further research is needed to elucidate the detailed mechanisms underlying its beneficial effects. Future investigations could focus on elucidating the molecular pathways involved in growth promotion by *B. licheniformis YB06* as well as their regulation in response to plant signals and environmental cues.

### 4.2. Identification of Growth-Promoting Genes Associated with YB06 via Genome-Wide Analysis

The genus *Bacillus* is characterized by a wide range of genomic G+C content, spanning from 34–35% in *B. cereus* and related species to 44–46% in *B. subtilis* and its close relatives. The genome size of *Bacillus* spp. also varies considerably, ranging from 3.7 to 6.4 Mb. Notably, the genome size and G+C content of *B. licheniformis* YB06 fall within the range observed for other sequenced *Bacillus* spp. [58]. Phylogenetic analyses based on 16S rRNA gene sequences and whole-genome comparisons consistently clustered *B. licheniformis* YB06 with the reference strain *B. licheniformis* ATCC14580. Comparative genomic analysis further corroborated this close relationship, revealing the highest degree of genetic similarity between *B. licheniformis* YB06 and *B. licheniformis* ATCC14580. Notably, *B. licheniformis* ATCC14580 has been extensively employed in the industrial production of antimicrobial enzymes [59]. Moreover, numerous studies have documented the capacity of *B. licheniformis* to synthesize various industrial enzymes and to serve as an efficient platform for the production of industrial commodities such as starch [17,60,61]. These findings collectively suggest that *B. licheniformis* YB06 may harbor significant potential for industrial applications. Comparative genomic analysis revealed the presence of structural variations (SVs) between the genomes of *B. licheniformis* YB06 and the reference strain *B. licheniformis* ATCC14580. Such SVs have been implicated in functional divergence between bacterial strains. This observation provides a genetic basis for the observed phenotypic differences [62]. Other *B. licheniformis* strains employed in evolutionary analyses, such as *B. licheniformis* MTB06, have been reported to produce bioactive compounds and contribute significantly to the development of flavor compounds in distilled spirits [59]. *B. licheniformis* DSM13 has been shown to synthesize a diverse array of industrial enzymes [63]. Additionally, *B. licheniformis* SRCM100141 is capable of producing various secondary metabolites with antimicrobial and plant growth-promoting activities [64]. Notably, *B. sonorensis*, a close relative of *B. licheniformis* based on 16S rRNA phylogenetic analysis, encompasses several strains that have demonstrated plant growth-promoting and biocontrol properties [65,66,67]. To gain deeper insights into the biological functions of *B. licheniformis* YB06, we conducted an in-depth annotation of its key metabolic pathways, particularly those associated with growth promotion and central carbon metabolism. These pathways encompass glycolysis, pyruvate oxidation, the pentose phosphate pathway, and gluconeogenesis [68]. Our analysis of the carbon metabolism pathways revealed that *B. licheniformis* YB06 is capable of utilizing a variety of carbohydrates, including glucose, fructose, xylulose, and ribulose. While no complete nitrogen fixation gene clusters (*nif*, *anf*, or *vnf*) were identified in the genome of *B. licheniformis* YB06, the strain exhibited growth on nitrogen-free medium, suggesting potential nitrogen-fixing capabilities. One hypothesis is that the nitrogen fixation genes reside on a plasmid, as reported in some diazotrophic bacteria [69]. However, further investigation is required to confirm this, for instance, through the detection of nitrogenase activity or its products. Alternative explanations, such as the utilization of trace nitrogen sources in the medium or other metabolic pathways, should also be considered. The biosynthesis of indole-3-acetic acid (IAA) in plant growth-promoting rhizobacteria (PGPR), including *Bacillus* spp., can occur through five distinct tryptophan-dependent pathways: the indole-3-acetamide (IAM), indole-3-acetonitrile (IAN), tryptophan side-chain oxidase (TSO), tryptamine (TAM), and indole-3-pyruvic acid (IPyA) pathways [70,71,72]. In the tryptophan metabolism pathway of *B. licheniformis* YB06, key enzymes involved in the indole-3-acetamide (IAM) and indole-3-acetaldehyde (IAAld) pathways were identified, namely, amidase and aldehyde dehydrogenase, respectively. These enzymes catalyze the hydrolysis of IAM and IAAld to IAA. The presence of other IAA-related enzymes, such as aldehyde dehydrogenase, catalase, and acyltransferase, further supports the IAA-producing capacity of *B. licheniformis* YB06, which was confirmed experimentally. This observation is consistent with numerous studies demonstrating the IAA production capabilities of *B. licheniformis* [37,57]. *B. licheniformis* YB06 possesses a complete repertoire of genes for the biosynthesis of 14 amino acids, including tryptophan. This finding aligns with previous research demonstrating the robust amino acid metabolism capabilities of *B. licheniformis*, highlighting its potential as an industrial strain for enzyme production [60,73,74]. Moreover, these amino acids are implicated in the accumulation of secondary metabolites, have enhanced tolerance to adverse environmental conditions, and serve as precursors or intermediates for the synthesis of numerous crucial compounds [75]. For instance, arginine acts as a precursor for polyamines, which play a pivotal role in regulating biological development [76], while tryptophan serves as the primary substrate for IAA biosynthesis [77]. Multiple siderophore synthesis-related genes were identified in *B. licheniformis* YB06, such as entA, entB, and entC, which are key genes for the production of high-efficiency siderophores in Gram-negative bacteria [78]. This is consistent with the research results of Nigris et al. [79], who also found key siderophore synthesis genes in *B. licheniformis*, proving its ability to produce siderophores. Flagella, the primary organelles responsible for bacterial motility, facilitate the movement of bacteria towards favorable growth environments and subsequent colonization [80]. Motility has been recognized as a crucial factor in bacterial colonization [81]. Genomic analysis of *B. licheniformis* YB06 revealed the presence of numerous genes associated with motility, suggesting that this strain may exhibit chemotactic behavior and possess motility capabilities. Furthermore, genes encoding proteins involved in motility, root colonization, matrix structure, and their regulation were identified within the *B. licheniformis* YB06 genome. The presence of flagellar and swarming motility-related proteins in *B. licheniformis* YB06 enables the bacteria to seek out and establish themselves in suitable environments [82]. Additionally, these proteins may contribute to the induction of host defense responses and facilitate nutrient acquisition [83]. The collective presence of these genes is likely to significantly enhance host-plant perception and recognition, thereby facilitating efficient root colonization and the establishment of a conducive microenvironment. This, in turn, would enable *B. licheniformis* YB06 to successfully colonize the plant rhizosphere and exert its plant growth-promoting effects.

### 4.3. Effects of Different Fertilization Conditions on Soil Physicochemical Properties and Bacterial Microbial Diversity

To investigate the potential of *B. licheniformis* YB06 as a biofertilizer for enhancing crop growth and yield, we conducted a study examining the effects of various fertilization regimes on the structure and diversity of the rhizosphere soil bacterial community associated with *C. pilosula*. A composite biofertilizer formulation with *B. licheniformis* YB06 as the primary active ingredient was employed, alongside commonly used agricultural fertilizers (chemical fertilizers, nitrogen fertilizers, and biofertilizers) applied individually and in combination. This study aimed to assess the efficacy of *B. licheniformis* YB06-based biofertilizer, both alone and in conjunction with other amendments, and to compare its performance with conventional fertilization practices. Our findings revealed significant differences in soil physicochemical properties among the various fertilization treatments (*p* < 0.05). Notably, the impact of these treatments on soil pH was minimal compared to other physicochemical parameters. This observation may be attributed to the inherent buffering capacity of the soil, which maintains a slightly alkaline pH. The short-term stability of soil pH under different fertilization regimes suggests that these conditions may be conducive to microbial colonization and plant growth [84]. All fertilization treatments significantly increased soil salinity (*p* < 0.05), with the most pronounced effect observed in the chemical fertilizer treatment. While moderate salinity levels can benefit plant growth, the substantial increase in salinity induced by chemical fertilizer indicates a marked short-term enrichment of soil inorganic salts, potentially promoting plant growth. However, prolonged application of chemical fertilizers may lead to excessive salt accumulation, resulting in soil salinization and detrimental effects on plant growth [85]. The elevated salinity levels also corresponded to increased soil electrical conductivity, as the concentration of dissolved salts directly influences this parameter. Notably, a moderate increase in electrical conductivity, within a certain range, has been associated with enhanced plant growth [86]. Furthermore, no significant differences in soil moisture content were observed among the treatment groups (*p* > 0.05), suggesting that short-term fertilization, while altering various soil physicochemical properties, did not significantly impact soil water retention capacity. However, previous research has demonstrated that long-term fertilization practices can exert distinct and substantial effects on soil water-holding capacity. For instance, Zhou et al. [87] reported that prolonged application of inorganic fertilizers alone did not significantly affect soil water retention, whereas the incorporation of organic amendments (e.g., wheat straw, pig manure, or cow manure) in combination with inorganic fertilizers significantly increased the available water capacity of the soil. Recent studies have further highlighted the potential of novel waste materials, integrating organic amendments and new materials with chemical fertilizers, to effectively enhance soil water retention [88,89,90]. In contrast to the chemical fertilizer treatment, the application of organic fertilizer, biofertilizer, or their combinations with chemical fertilizer resulted in elevated soil pH, moisture content, total nitrogen (TN), total potassium (TK), and soil organic matter (SOM). These findings underscore the superior efficacy of integrated organic and/or biofertilizer amendments over sole chemical fertilizer application in enhancing soil fertility. Previous research has demonstrated that the long-term application of organic fertilizers or combined organic–microbial fertilizers can stimulate microbial biomass and enzymatic activity, thereby improving both the quantity and quality of SOM [91,92]. The combined application of organic and chemical fertilizers resulted in higher levels of soil total nitrogen (TN), available nitrogen (AN), total phosphorus (TP), and soil organic matter (SOM) compared to the combination of organic fertilizer and biofertilizer. This observation suggests that, under short-term fertilization regimes, the co-application of organic and chemical fertilizers exerts a more pronounced effect on soil physicochemical properties, while biofertilizers alone may not fully substitute for chemical fertilizers in the short term. Microorganisms play a pivotal role in nitrogen and phosphorus cycling within the soil ecosystem [93,94]. Chen et al. [8] reported that *B. licheniformis* SL-44, a component of microbial carbon-based preparations (BCMs), can enhance plant photosynthetic capacity and endogenous phytohormone biosynthesis, thereby increasing the uptake of total nitrogen (TN), total phosphorus (TP), and total potassium (TK). This observation may partially explain the lower TN, AN, TP, and SOM levels observed in our study following the combined application of biofertilizer and bacterial agents, compared to the combination of chemical and biofertilizers. Conversely, the higher available phosphorus (AP) content in the biofertilizer and bacterial agent treatment can be attributed to the phosphate-solubilizing activity of the functional bacteria, which convert insoluble soil phosphorus into plant-available forms [95,96,97]. This finding aligns with the recent discovery of James et al. [98], who isolated a phosphate-solubilizing strain of *B. licheniformis* NJ04, which facilitates plant utilization of insoluble soil phosphate. Gomez-Ramirez and Uribe-Velez [99] similarly observed that their selected *Bacillus* strains exhibited tricalcium phosphate solubilization, phytate mineralization, and phosphate release from rice straw (RS) in vitro, resulting in a two-fold increase in plant phosphate uptake. Taken together, our findings highlight the potential of biofertilizers to enhance the bioavailability of nitrogen and phosphorus, thereby promoting plant growth. However, further research is warranted to elucidate the long-term impacts of combined biofertilizer and organic fertilizer application on soil physicochemical properties.

The application of different fertilizers resulted in a significant increase in the number of operational taxonomic units (OTUs) across various taxonomic levels (phylum, class, order, family, and genus), with the highest OTU richness observed in the combined organic fertilizer and biofertilizer treatment. This finding suggests a high degree of compatibility between biofertilizers and organic fertilizers, with their synergistic interaction promoting soil bacterial diversity. Lili et al. [100] reported that the combined application of bio-organic fertilizers and microbial agents significantly enhanced tomato growth, yield, and quality, particularly during the mid-to-late fruiting stages. Similarly, Liu et al. [101] demonstrated that the combined use of biocontrol agents L-25 and L-9 with organic fertilizers effectively suppressed bacterial wilt disease by modulating the soil microbial community structure. The analysis of soil bacterial α-diversity under different fertilization regimes revealed that organic fertilizer application enhanced the resilience of soil microbial communities compared to chemical fertilization alone. The addition of CFOF (chemical fertilizer + organic fertilizer) and NF (nitrogen fertilizer) significantly increased bacterial diversity and richness. However, the OFBOF (organic fertilizer + biofertilizer) treatment exhibited a significant decrease in both diversity and richness. This reduction may be attributed to the dominance of the introduced probiotic bacteria, which could outcompete native species for resources and space or potentially exert antagonistic effects on certain bacterial taxa, thereby diminishing overall community diversity and richness [102]. The principal coordinate analysis (PCoA) revealed that fertilization regimes significantly altered the bacterial community structure within the *C. pilosula* rhizosphere. Comparison of the different fertilization treatments indicated that the use of bio-organic fertilizer was the determining factor in driving shifts in the rhizosphere soil bacterial community composition. Across all fertilization treatments, *Acidobacteriota*, *Proteobacteria*, *Gemmatimonadota*, *Actinobacteria*, and *Chloroflexi* emerged as the predominant bacterial phyla. Notably, the relative abundance of *Deitaproteobacteria* (a class within *Proteobacteria*) increased most significantly following the combined application of biofertilizer and organic fertilizer. Previous studies have identified *Proteobacteria*, *Actinobacteria*, and *Firmicutes* as the dominant plant growth-promoting bacterial groups in soils cultivated with cucumber, corn, and ryegrass [103]. Additionally, del Barrio-Duque et al. [104] reported that interactions between endophytic *Proteobacteria* strains and *Serendipita* indica enhanced biocontrol activity against fungal pathogens. Members of the *Chloroflexi* phylum are known to participate in organic matter decomposition, nitrogen removal, and biofilm formation, with their roles varying depending on environmental conditions [105]. *Acidobacteriota*, a phylum of slow-growing oligotrophic bacteria, are often suppressed by the increased nutrient availability resulting from the application of microbial agents [106]. Conversely, *Actinobacteria*, a group of copiotrophic bacteria that thrive in nutrient-rich environments, exhibited the highest abundance under nitrogen fertilizer treatment, likely due to the increased nitrogen and carbon availability [107]. The observed enrichment of other potentially beneficial microorganisms in the *C. pilosula* rhizosphere may be attributed to the suppression of soil-borne pathogens and the re-establishment of a balanced microbial community structure. Numerous studies have reported that the dynamics of rhizosphere microbial communities are influenced by various soil properties, such as soil pH, nitrogen, and water conditions [108]. Our study revealed a positive correlation between soil pH and bacterial communities, with near-neutral soil pH suggesting that the application of organic fertilizer can mitigate soil acidification. Soil AN, pH, TN, and TP emerged as the most critical environmental factors affecting the abundance of bacterial phyla in the rhizosphere. Consequently, key management and monitoring indicators for fertilization should prioritize the regulation of AN, pH, TN, and TP. Both existing research and our experimental results demonstrate that biofertilizers, particularly those containing *B. licheniformis* as the functional microbe, can modulate soil pH, enhance the release of inorganic salts from fertilizers, and improve nutrient uptake by plants. This, in turn, modifies the AN, TN, and TP indices in the soil and influences the abundance of rhizosphere bacteria.

## 5. Conclusions

This study focused on *B. licheniformis* YB06, a potential plant growth-promoting rhizobacterium (PGPR) isolated from the rhizosphere soil of healthy *C. pilosula plants*. We employed a multi-faceted approach, including phenotypic characterization, genomic analysis, and experiments, to investigate its growth-promoting capabilities and the underlying mechanisms. Our findings demonstrate that *B. licheniformis* YB06 is a potent PGPR with multiple growth-promoting mechanisms, including the production of indole-3-acetic acid (IAA), siderophores, and 1-aminocyclopropane-1-carboxylate (ACC) deaminase, as well as the ability to fix nitrogen. These results highlight the potential of *B. licheniformis* YB06 as a biofertilizer for enhancing crop growth and development. Furthermore, we explored the effects of different fertilization regimes, incorporating a *B. licheniformis* YB06-based biofertilizer alongside organic and chemical fertilizers, on the diversity and composition of the *C. pilosula* rhizosphere microbiome. Our results revealed distinct impacts of various fertilization practices on soil physicochemical properties and microbial diversity, providing valuable insights into the complex interactions between microbial communities and agricultural management practices and contributing to a deeper understanding of the intricate responses of microbial diversity to microbial inoculants.

## Figures and Tables

**Table 1:** Selected plant growth-promoting pathways and genes.

Pathways	Function	Genes	Details of Genes
Carbon metabolism	The glycolysis cycle	*pyk*	pyruvate kinase [EC:2.7.1.40]
	Organic acid synthesis	*ackA*	acetate kinase [EC:2.7.2.1]
		*fumC*	fumarate hydratase, class II [EC:4.2.1.2]
		*gltA*	citrate synthase [EC:2.3.3.1]
		*aceA*	isocitrate lyase [EC:4.1.3.1]
		*acnA*	aconitate hydratase [EC:4.2.1.3]
		*mdh*	malate dehydrogenase [EC:1.1.1.37]
		*sdhB*	succinate dehydrogenase [EC:1.3.5.1 1.3.5.4]
Nitrogen metabolism	Nitrogen metabolism	*arcC*	carbamate kinase [EC:2.7.2.2]
		*gudB*	glutamate dehydrogenase [EC:1.4.1.2]
		*gltB*	glutamate synthase (NADPH) large chain [EC:1.4.1.13]
		*glnA*	glutamine synthetase [EC:6.3.1.2]
		*nirB*	nitrite reductase (NADH) large subunit [EC:1.7.1.15]
		*narG* *narZ* *nxrA*	nitrate reductase nitrite oxidoreductase, alpha subunit [EC:1.7.5.1 1.7.99.-]
		*norB*	nitric oxide reductase subunit B [EC:1.7.2.5]
		*ncd2* *npd*	nitronate monooxygenase [EC:1.13.12.16]
		*nirD*	nitrite reductase (NADH) small subunit [EC:1.7.1.15]
Tryptophan metabolism	Tryptophan metabolism	*ALDH*	aldehyde dehydrogenase (NAD+) [EC:1.2.1.3]
		*amiE*	amidase [EC:3.5.1.4]
		*katE*	catalase [EC:1.11.1.6]
		*atoB*	acetyl-CoA C-acetyltransferase [EC:2.3.1.9]
Biosynthesis of siderophore group nonribosomal peptides	Biosynthesis of siderophore group nonribosomal peptides	*entA*	2,3-dihydro-2,3-dihydroxybenzoate dehydrogenase [EC:1.3.1.28]
		*entB*	bifunctional isochorismate lyase/ aryl carrier protein [EC:3.3.2.1 6.3.2.14]
		*entC*	isochorismate synthase [EC:5.4.4.2]
		*mbtH*	nocI; MbtH protein
Flagellar assembly	Flagellar assembly	*flhA*	flagellar biosynthesis protein FlhA
		*fliD*	flagellar hook-associated protein 2
		*fliI*	flagellum-specific ATP synthase [EC:7.4.2.8]
		*fliF*	flagellar M-ring protein FliF
		*fliD*	flagellar hook-associated protein 2
	Flagellar motor proteins	*motA*	chemotaxis protein MotA
		*motB*	chemotaxis protein MotB
Bacterial chemotaxis	Bacterial chemotaxis	*mcp*	methyl-accepting chemotaxis protein
		*cheR*	chemotaxis protein methyltransferase CheR [EC:2.1.1.80]
		*cheA*	chemotaxis family, sensor kinase CheA [EC:2.7.13.3]

**Table 2:** Physicochemical characteristics of soil under different fertilization conditions.

Group	Hydration (%)	pH	EC (μs/cm)	Salinity (mol/L)	TN (mg/kg)	AN (mg/kg)	TP (mg/kg)	AP (mg/kg)	TK (mg/kg)	AK (mg/kg)	SOM (mg/kg)
**CF**	9.31 ± 0.54 ^c^	7.02 ± 0.09 ^c^	377.33 ± 9.45 ^a^	0.051 ± 0.04 ^a^	103.51 ± 6.25 ^c^	86 ± 0.19 ^b^	280.83 ± 7.04 ^a^	0.18 ± 0.07 ^a^	23.02 ± 2.3 ^a^	0.16 ± 0.05 ^a^	2.90 ± 0.27 ^b^
**OFCF**	10.47 ± 0.26 ^b^	7.23 ± 0.08 ^b^	329.33 ± 5.03 ^b^	0.037 ± 0.02 ^a^	139.70 ± 5.73 ^b^	89.33 ± 7.57 ^b^	235.00 ± 25.92 ^b^	0.17 ± 0.04 ^a^	25.94 ± 1.13 ^a^	0.12 ± 0.001 ^a^	5.09 ± 0.78 ^a^
**NF**	9.81 ± 0.13 ^bc^	7.47 ± 0.04 ^a^	295 ± 3 ^c^	0.025 ± 0.0 ^a^	191.01 ± 12.41 ^a^	119.67 ± 6.03 ^a^	91.46 ± 2.87 ^d^	0.24 ± 0.12 ^a^	25.69 ± 0.46 ^a^	0.104 ± 0.058 ^a^	3.31 ± 0.19 ^b^
**OF** **BOF**	11.67 ± 0.6 ^a^	7.41 ± 0.02 ^ab^	241 ± 7 ^d^	0.029 ± 0.01 ^a^	121.13 ± 0.04 ^bc^	79.67 ± 8.08 ^b^	133.68 ± 3.75 ^c^	0.28 ± 0.03 ^a^	26.34 ± 6.32 ^a^	0.1 ± 0.035 ^a^	5.58 ± 0.1 ^a^

Note: Values within a column followed by different letters indicate significant differences (*p* < 0.05) according to Tukey’s HSD test. CF: chemical fertilizer; OFCF: organic and chemical fertilizer; NF: nitrogen fertilizer; OFBOF: organic fertilizer and biofertilizer; EC: electrical conductivity; TN: total nitrogen; AN: available nitrogen; TP: total phosphorus; AP: available phosphorus; TK: total potassium; AK: available potassium; SOM: soil organic matter.

**Table 3:** Summary of bacterial OTU statistics.

Sample	Phylum	Class	Order	Family	Genus	Species
CF	28	84	167	232	342	268
CFOF	28	77	154	219	321	252
OFBOF	28	85	171	235	348	275
NF	29	84	162	220	321	253

## Data Availability

All data generated during this study are included in this published article and its Supplementary Materials. Additionally, the bacterial genome assembly data are available in the NCBI GenBank database under accession number CP102663 (*Bacillus* sp. BAC chromosome, complete genome–nucleotide–NCBI (https://www.nih.gov/), accessed on 1 March 2024).

## Supplementary Materials

The following supporting information can be downloaded at https://www.mdpi.com/article/10.3390/microorganisms12091861/s1, Figure S1: Influence of various factors on the growth of *B. licheniformis* YB06: (A) pH, (B) temperature, (C) NaCl concentration; Figure S2: Effect of pH (A), temperature (B), and NaCl concentration (C) on indole-3-acetic acid (IAA) production by the strain. Effect of pH (D), temperature (E), and NaCl concentration (F) on nitrogen fixation capacity of the strain. Effect of pH (G), temperature (H), and NaCl concentration (I) on siderophore production by the strain; Figure S3. Effects of varying concentrations of *B. licheniformis* YB06 (CFU/mL) on the (A) germination status of *C. pilosula* seeds after 14 days of cultivation (50 seeds per treatment, and ungerminated seeds were removed), (B) germination rate of seeds after 14 days of cultivation, and (C) germination potential of seeds after 14 days of cultivation. Effects of varying concentrations of *B. licheniformis* YB06 on the (D) root length and (E) fresh weight of *C. pilosula* seedlings. This was a preliminary experiment with one treatment group per concentration; Figure S4: (A) Top 20 KEGG pathways identified in the *B. licheniformis* YB06 genome. (B) Gene Ontology (GO) annotation of the complete genome of *B. licheniformis* YB06; Figure S5: (A) Classification of carbohydrate-active enzymes (CAZymes) identified in the genome of *B. licheniformis* YB06. (B) Predicted secondary metabolite gene clusters in the genome of *B. licheniformis* YB06; Figure S6: Carbon metabolism pathways in *B. licheniformis* YB06; Figure S7: Amino acid metabolism pathways in *B. licheniformis YB06*; Figure S8: Biosynthesis of siderophore group nonribosomal peptides in *B. licheniformis YB06*; Figure S9: Flagellar assembly pathway in *B. licheniformis* YB06; Figure S10: Chemotaxis-related proteins and gene pathways in *B. licheniformis* YB06; Table S1: Summary statistics of non-coding RNA prediction results; Table S2: Statistics of gene island prediction results; Table S3: Summary of predicted prophages; Table S4: Summary of identified virulence factors; Table S5. Summary of identified virulence factors.

## References

[1] Luan F., Ji Y., Peng L., Liu Q., Cao H., Yang Y., He X., Zeng N. (2021). Extraction, purification, structural characteristics and biological properties of the polysaccharides from *Codonopsis pilosula*: A review. Carbohydr. Polym..

[2] Guo H., Lou Y., Hou X., Han Q., Guo Y., Li Z., Guan X., Liu H., Zhang C. (2024). A systematic review of the mechanism of action and potential medicinal value of *Codonopsis pilosula* in diseases. Front. Pharmacol..

[3] Zou Y.-F., Zhang Y.-Y., Paulsen B.S., Fu Y.-P., Huang C., Feng B., Li L.-X., Chen X.-F., Jia R.-Y., Song X. (2020). Prospects of *Codonopsis pilosula* polysaccharides: Structural features and bioactivities diversity. Trends Food Sci. Technol..

[4] Lv B., Yang X., Xue H., Nan M., Zhang Y., Liu Z., Bi Y., Shang S. (2023). Isolation of main pathogens causing postharvest disease in fresh *Codonopsis pilosula* during different storage stages and ozone control against disease and mycotoxin accumulation. J. Fungi.

[5] Vasco C., Torres B., Jácome E., Torres A., Eche D., Velasco C. (2021). Use of chemical fertilizers and pesticides in frontier areas: A case study in the Northern Ecuadorian Amazon. Land Use Policy.

[6] Cetin M., Aljama A.M.O., Alrabiti O.B.M., Adiguzel F., Sevik H., Zeren Cetin I. (2022). Using topsoil analysis to determine and map changes in Ni Co pollution. Water Air Soil Pollut..

[7] Ma J., Chen Y., Wang K., Huang Y., Wang H. (2021). Re-utilization of Chinese medicinal herbal residues improved soil fertility and maintained maize yield under chemical fertilizer reduction. Chemosphere.

[8] Chen W., Wu Z., Liu C., Zhang Z., Liu X. (2023). Biochar combined with Bacillus subtilis SL-44 as an eco-friendly strategy to improve soil fertility, reduce Fusarium wilt, and promote radish growth. Ecotoxicol. Environ. Saf..

[9] Ha-Tran D.M., Nguyen T.T.M., Hung S.-H., Huang E., Huang C.-C. (2021). Roles of plant growth-promoting rhizobacteria (PGPR) in stimulating salinity stress defense in plants: A review. Int. J. Mol. Sci..

[10] Wang J., He Y., Li T., Li C., Xu X., Xiang H., Wang X., Wu Z. (2022). Complex biochemical synergistic interactions between two rhizobacteria grown in consortium, *Bacillus subtilis* SL-44 and *Enterobacter hormaechei* Wu-15. Rhizosphere.

[11] Vocciante M., Grifoni M., Fusini D., Petruzzelli G., Franchi E. (2022). The role of plant growth-promoting rhizobacteria (PGPR) in mitigating plant’s environmental stresses. Appl. Sci..

[12] Ali S., Hameed S., Shahid M., Iqbal M., Lazarovits G., Imran A. (2020). Functional characterization of potential PGPR exhibiting broad-spectrum antifungal activity. Microbiol. Res..

[13] Liu Y., Zhang W., Zhang Z., Kou Z., Wang X., Wang Y., Su X., Zhang J., Liu L., Yan F. (2024). Biocontrol effects of three antagonistic bacteria strains against *Codonopsis pilosula* wilt disease caused by *Fusarium oxysporum*. Biol. Control..

[14] Feng M.-Q., Feng Y.-C., Gou W. (2023). Identification of the pathogen of *Codonopsis pilosula* stem base rot and screening of its control agents. J. Fungi.

[15] Verma J., Kumar C., Sharma M., Shukla A.C., Saxena S. (2024). Exploitation of microbial consortia for formulating biofungicides, biopesticides, and biofertilizers for plant growth promotion. Entrepreneurship with Microorganisms.

[16] Samada L.H., Tambunan U.S.F. (2020). Biopesticides as promising alternatives to chemical pesticides: A review of their current and future status. Online J. Biol. Sci..

[17] Muras A., Romero M., Mayer C., Otero A. (2021). Biotechnological applications of *Bacillus licheniformis*. Crit. Rev. Biotechnol..

[18] Arnaouteli S., Bamford N.C., Stanley-Wall N.R., Kovács Á.T. (2021). Bacillus subtilis biofilm formation and social interactions. Nat. Rev. Microbiol..

[19] Miljaković D., Marinković J., Balešević-Tubić S. (2020). The significance of *Bacillus* spp. in disease suppression and growth promotion of field and vegetable crops. Microorganisms.

[20] Wang Q., Ou E.-L., Wang P.-C., Chen Y., Wang Z.-Y., Wang Z.-W., Fang X.-W., Zhang J.-L. (2022). Bacillus amyloliquefaciens GB03 augmented tall fescue growth by regulating phytohormone and nutrient homeostasis under nitrogen deficiency. Front. Plant Sci..

[21] Han X., Shen D., Xiong Q., Bao B., Zhang W., Dai T., Zhao Y., Borriss R., Fan B. (2021). The plant-beneficial rhizobacterium Bacillus velezensis FZB42 controls the soybean pathogen *Phytophthora sojae* due to bacilysin production. Appl. Environ. Microbiol..

[22] Lane D. (1991). 16S/23S rRNA Sequencing. Nucleic Acid Techniques in Bacterial Systematics.

[23] Shahab S., Ahmed N., Khan N.S. (2009). Indole acetic acid production and enhanced plant growth promotion by indigenous PSBs. Afr. J. Agric. Res..

[24] Payne S.M. (1994). [25] Detection, isolation, and characterization of siderophores. Methods Enzymol..

[25] Li Z., Chang S., Lin L., Li Y., An Q. (2011). A colorimetric assay of 1-aminocyclopropane-1-carboxylate (ACC) based on ninhydrin reaction for rapid screening of bacteria containing ACC deaminase. Lett. Appl. Microbiol..

[26] Meddeb-Mouelhi F., Moisan J.K., Beauregard M. (2014). A comparison of plate assay methods for detecting extracellular cellulase and xylanase activity. Enzym. Microb. Technol..

[27] de Veras B.O., dos Santos Y.Q., Diniz K.M., Carelli G.S.C., dos Santos E.A. (2018). Screening of protease, cellulase, amylase and xylanase from the salt-tolerant and thermostable marine Bacillus subtilis strain SR60. F1000Research.

[28] Fasiku S.A., Ogunsola O.F., Fakunle A., Olanbiwoninu A.A. (2020). Isolation of bacteria with potential of producing extracellular enzymes (Amylase, Cellulase and Protease) from soil samples. J. Adv. Microbiol..

[29] Schollenberger C. (1945). Determination of soil organic matter. Soil Sci..

[30] Kirk P.L. (1950). Kjeldahl method for total nitrogen. Anal. Chem..

[31] Rowland A., Haygarth P. (1997). Determination of Total Dissolved Phosphorus in Soil Solutions.

[32] Broderick E., Zack P. (1951). Flame Spectrophotometry for Determination of Sodium, Potassium, and Lithium in Glass. Anal. Chem..

[33] Chen S., Lin B., Li Y., Zhou S. (2020). Spatial and temporal changes of soil properties and soil fertility evaluation in a large grain-production area of subtropical plain, China. Geoderma.

[34] Koetsier G., Cantor E. (2019). A practical guide to analyzing nucleic acid concentration and purity with microvolume spectrophotometers. N. Engl. Biolabs Inc.

[35] Saxena A.K., Kumar M., Chakdar H., Anuroopa N., Bagyaraj D. (2020). Bacillus species in soil as a natural resource for plant health and nutrition. J. Appl. Microbiol..

[36] de O., Nunes P.S., De Medeiros F.H., De Oliveira T.S., de Almeida Zago J.R., Bettiol W. (2023). Bacillus subtilis and *Bacillus licheniformis*promote tomato growth. Braz. J. Microbiol..

[37] Bhutani N., Maheshwari R., Sharma N., Kumar P., Dang A.S., Suneja P. (2022). Characterization of halo-tolerant plant growth promoting endophytic *Bacillus licheniformis*MHN 12. J. Genet. Eng. Biotechnol..

[38] Devi S., Sharma S., Tiwari A., Bhatt A.K., Singh N.K., Singh M., Kaushalendra, Kumar A. (2023). Screening for multifarious plant growth promoting and biocontrol attributes in bacillus strains isolated from indo gangetic soil for enhancing growth of rice crops. Microorganisms.

[39] Liu J., Zhang J., Zhu M., Wan H., Chen Z., Yang N., Duan J., Wei Z., Hu T., Liu F. (2022). Effects of plant growth promoting rhizobacteria (PGPR) strain *Bacillus licheniformis* with biochar amendment on potato growth and water use efficiency under reduced irrigation regime. Agronomy.

[40] Wan G.-Z., Wang L., Jin L., Chen J. (2021). Evaluation of environmental factors affecting the quality of *Codonopsis pilosula* based on chromatographic fingerprint and MaxEnt model. Ind. Crops Prod..

[41] Yan H., He J., Xu X., Yao X., Wang G., Tang L., Feng L., Zou L., Gu X., Qu Y. (2021). Prediction of potentially suitable distributions of *Codonopsis pilosula* in China based on an optimized MaxEnt model. Front. Ecol. Evol..

[42] Ahmad E., Sharma P.K., Khan M.S. (2022). IAA biosynthesis in bacteria and its role in plant-microbe interaction for drought stress management. Plant Stress Mitigators: Action and Application.

[43] Wagi S., Ahmed A. (2019). *Bacillus* spp.: Potent microfactories of bacterial IAA. PeerJ.

[44] Sansinenea E. (2019). *Bacillus* spp.: As plant growth-promoting bacteria. Secondary Metabolites of Plant Growth Promoting Rhizomicroorganisms: Discovery and Applications.

[45] Shah R., Amaresan N., Patel P., Jinal H.N., Krishnamurthy R. (2020). Isolation and characterization of *Bacillus* spp. endowed with multifarious plant growth-promoting traits and their potential effect on tomato (*Lycopersicon esculentum*) seedlings. Arab. J. Sci. Eng..

[46] Choo Q.-C., Samian M.-R., Najimudin N. (2003). Phylogeny and characterization of three nifH-homologous genes from *Paenibacillus azotofixans*. Appl. Environ. Microbiol..

[47] Masood S., Zhao X.Q., Shen R.F. (2020). Bacillus pumilus promotes the growth and nitrogen uptake of tomato plants under nitrogen fertilization. Sci. Hortic..

[48] Groß C., Hossen S., Hartmann H., Noll M., Borken W. (2022). Biological nitrogen fixation and nifH gene abundance in deadwood of 13 different tree species. Biogeochemistry.

[49] Krewulak K.D., Vogel H.J. (2008). Structural biology of bacterial iron uptake. Biochim. Biophys. Acta (BBA)-Biomembr..

[50] Parmar H.Y., Chakraborty H. (2016). Effect of siderophore on plant growth promotion. Int. J. Appl. Pure Sci. Agric..

[51] Naing A.H., Maung T.T., Kim C.K. (2021). The ACC deaminase-producing plant growth-promoting bacteria: Influences of bacterial strains and ACC deaminase activities in plant tolerance to abiotic stress. Physiol. Plant..

[52] Misra S., Chauhan P.S. (2020). ACC deaminase-producing rhizosphere competent *Bacillus* spp. mitigate salt stress and promote *Zea mays* growth by modulating ethylene metabolism. 3 Biotech..

[53] Shahid M., Singh U.B., Khan M.S., Singh P., Kumar R., Singh R.N., Kumar A., Singh H.V. (2023). Bacterial ACC deaminase: Insights into enzymology, biochemistry, genetics, and potential role in amelioration of environmental stress in crop plants. Front. Microbiol..

[54] Gowtham H., Singh B., Murali M., Shilpa N., Prasad M., Aiyaz M., Amruthesh K., Niranjana S. (2020). Induction of drought tolerance in tomato upon the application of ACC deaminase producing plant growth promoting rhizobacterium Bacillus subtilis Rhizo SF 48. Microbiol. Res..

[55] Mukhtar T., Rehman S.U., Smith D., Sultan T., Seleiman M.F., Alsadon A.A., Amna, Ali S., Chaudhary H.J., Solieman T.H. (2020). Mitigation of heat stress in *Solanum lycopersicum* L. by ACC-deaminase and exopolysaccharide producing Bacillus cereus: Effects on biochemical profiling. Sustainability.

[56] Kulkova I., Dobrzyński J., Kowalczyk P., Bełżecki G., Kramkowski K. (2023). Plant growth promotion using *Bacillus cereus*. Int. J. Mol. Sci..

[57] Medison R.G., Jiang J., Medison M.B., Tan L.-T., Kayange C.D., Sun Z., Zhou Y. (2023). Evaluating the potential of *Bacillus licheniformis*YZCUO202005 isolated from lichens in maize growth promotion and biocontrol. Heliyon.

[58] Zeng Q., Xie J., Li Y., Gao T., Xu C., Wang Q. (2018). Comparative genomic and functional analyses of four sequenced Bacillus cereus genomes reveal conservation of genes relevant to plant-growth-promoting traits. Sci. Rep..

[59] Yang F., Liu Y., Chen L., Li J., Wang L., Du G. (2020). Genome sequencing and flavor compound biosynthesis pathway analyses of *Bacillus licheniformis*isolated from Chinese Maotai-flavor liquor-brewing microbiome. Food Biotechnol..

[60] Zhu J., Liu M., Kang J., Wang S., Zha Z., Zhan Y., Wang Z., Li J., Cai D., Chen S. (2024). Engineering *Bacillus licheniformis* as industrial chassis for efficient bioproduction from starch. Bioresour. Technol..

[61] He H., Yu Q., Ding Z., Zhang L., Shi G., Li Y. (2023). Biotechnological and food synthetic biology potential of platform strain: *Bacillus licheniformis*. Synth. Syst. Biotechnol..

[62] Guo J., Cao K., Deng C., Li Y., Zhu G., Fang W., Chen C., Wang X., Wu J., Guan L. (2020). An integrated peach genome structural variation map uncovers genes associated with fruit traits. Genome Biol..

[63] Veith B., Herzberg C., Steckel S., Feesche J., Maurer K.H., Ehrenreich P., Bäumer S., Henne A., Liesegang H., Merkl R. (2004). The complete genome sequence of *Bacillus licheniformis* DSM13, an organism with great industrial potential. J. Mol. Microbiol. Biotechnol..

[64] Harwood C.R., Mouillon J.-M., Pohl S., Arnau J. (2018). Secondary metabolite production and the safety of industrially important members of the Bacillus subtilis group. FEMS Microbiol. Rev..

[65] Thilagar G., Bagyaraj D., Podile A.R., Vaikuntapu P.R. (2018). Bacillus sonorensis, a novel plant growth promoting rhizobacterium in improving growth, nutrition and yield of chilly (*Capsicum annuum* L.). Proc. Natl. Acad. Sci. India Sect. B Biol. Sci..

[66] Deng J., Kong S., Wang F., Liu Y., Jiao J., Lu Y., Zhang F., Wu J., Wang L., Li X. (2020). Identification of a new Bacillus sonorensis strain KLBC GS-3 as a biocontrol agent for postharvest green mould in grapefruit. Biol. Control.

[67] Harinathan B., Sankaralingam S., Palpperumal S., Balachandran C., Hashem A., Alqarawi A.A., Abd_Allah E.F., Arokiyaraj S., Baskar K. (2021). Impact of rhizobacterium *Bacillus sonorensis* on propagation of Abelmoschus esculentus and its antimicrobial activity. J. King Saud Univ. -Sci..

[68] Baslam M., Mitsui T., Sueyoshi K., Ohyama T. (2020). Recent advances in carbon and nitrogen metabolism in C3 plants. Int. J. Mol. Sci..

[69] Nuti M., Lepidi A., Prakash R., Schilperoort R., Cannon F. (1979). Evidence for nitrogen fixation (nif) genes on indigenous Rhizobium plasmids. Nature.

[70] Ghosh S., Ghosh P., Maiti T. (2011). Production and metabolism of indole acetic acid (IAA) by root nodule bacteria (Rhizobium): A review. J. Pure Appl. Microbiol..

[71] Sun S.-L., Yang W.-L., Fang W.-W., Zhao Y.-X., Guo L., Dai Y.-J. (2018). The plant growth-promoting rhizobacterium Variovorax boronicumulans CGMCC 4969 regulates the level of indole-3-acetic acid synthesized from indole-3-acetonitrile. Appl. Environ. Microbiol..

[72] Chaudhary T., Gera R., Shukla P. (2021). Deciphering the potential of Rhizobium pusense MB-17a, a plant growth-promoting root endophyte, and functional annotation of the genes involved in the metabolic pathway. Front. Bioeng. Biotechnol..

[73] Cai D., Zhu J., Li Y., Li L., Zhang M., Wang Z., Yang H., Li J., Yang Z., Chen S. (2020). Systematic engineering of branch chain amino acid supply modules for the enhanced production of bacitracin from *Bacillus licheniformis*. Metab. Eng. Commun..

[74] Zhou W., Zeng S., Yu J., Xiang J., Zhang F., Takriff M.S., Ding G., Ma Z., Zhou X. (2023). Complete genome sequence of Bacillus Licheniformis NWMCC0046, a candidate for the laundry industry. J. Basic Microbiol..

[75] Paria P., Chakraborty H.J., Behera B.K. (2022). Identification of novel salt tolerance-associated proteins from the secretome of Enterococcus faecalis. World J. Microbiol. Biotechnol..

[76] Rakesh B., Sudheer W., Nagella P. (2021). Role of polyamines in plant tissue culture: An overview. Plant Cell Tissue Organ. Cult..

[77] Zhang B.-X., Li P.-S., Wang Y.-Y., Wang J.-J., Liu X.-L., Wang X.-Y., Hu X.-M. (2021). Characterization and synthesis of indole-3-acetic acid in plant growth promoting *Enterobacter* sp.. RSC Adv..

[78] Khazaal M.T., Faraag A.H., Hamada M.A., El-Hendawy H.H. (2024). Characterization and Statistical Optimization of Enterobatin Synthesized by Escherichia coli OQ866153. Biochem. Genet..

[79] Nigris S., Baldan E., Tondello A., Zanella F., Vitulo N., Favaro G., Guidolin V., Bordin N., Telatin A., Barizza E. (2018). Biocontrol traits of *Bacillus licheniformis* GL174, a culturable endophyte of Vitis vinifera cv. Glera. BMC Microbiol..

[80] Tans-Kersten J., Brown D., Allen C. (2004). Swimming motility, a virulence trait of Ralstonia solanacearum, is regulated by FlhDC and the plant host environment. Mol. Plant-Microbe Interact..

[81] Bowen G., Rovira A. (1976). Microbial colonization of plant roots. Annu. Rev. Phytopathol..

[82] Kobayashi K., Kanesaki Y., Yoshikawa H. (2016). Genetic analysis of collective motility of Paenibacillus sp. NAIST15-1. PLoS Genet..

[83] Chen X.H., Koumoutsi A., Scholz R., Eisenreich A., Schneider K., Heinemeyer I., Morgenstern B., Voss B., Hess W.R., Reva O. (2007). Comparative analysis of the complete genome sequence of the plant growth–promoting bacterium *Bacillus amyloliquefaciens* FZB42. Nat. Biotechnol..

[84] Chen J., Yang W., Li J., Anwar S., Wang K., Yang Z., Gao Z. (2021). Effects of herbicides on the microbial community and urease activity in the rhizosphere soil of maize at maturity stage. J. Sens..

[85] Machado R.M.A., Serralheiro R.P. (2017). Soil salinity: Effect on vegetable crop growth. Management practices to prevent and mitigate soil salinization. Horticulturae.

[86] Zhu Z., Zhang H., Leng J., Niu H., Chen X., Liu D., Chen Y., Gao N., Ying H. (2020). Isolation and characterization of plant growth-promoting rhizobacteria and their effects on the growth of *Medicago sativa* L. under salinity conditions. Antonie Van Leeuwenhoek.

[87] Zhou H., Chen C., Wang D., Arthur E., Zhang Z., Guo Z., Peng X., Mooney S.J. (2020). Effect of long-term organic amendments on the full-range soil water retention characteristics of a Vertisol. Soil Tillage Res..

[88] Lu H., Zhang Y., Tian T., Li X., Wu J., Yang H., Huang H. (2023). Preparation and properties of *Sanxan* gel based fertilizer for water retention and slow-release. Int. J. Biol. Macromol..

[89] Mekhzoum M.E.M., Aasfar A., Mzibra A., El Mernissi N., Farrie Y., Khouloud M., Boulif R., Qaiss A.e.k., Kadmiri I.M., Bouhfid R. (2023). Phosphorus fertilizer coated with polysaccharide-enriched extracts from the red seaweed Schizymenia dubyi for slow release and water retention. J. Appl. Phycol..

[90] Soliman E., Mansour M.M. (2024). Enhancing soil organic carbon content and water retention using polyvinyl alcohol cross-linked with chitosan and pectin. J. Soil Sci. Plant Nutr..

[91] Wei X., Xie B., Wan C., Song R., Zhong W., Xin S., Song K. (2024). Enhancing soil health and plant growth through microbial fertilizers: Mechanisms, benefits, and sustainable agricultural practices. Agronomy.

[92] Shao F., Tao W., Yan H., Wang Q. (2023). Effects of Microbial Organic Fertilizer (MOF) Application on Desert Soil Enzyme Activity and Jujube Yield and Quality. Agronomy.

[93] Tian J., Ge F., Zhang D., Deng S., Liu X. (2021). Roles of phosphate solubilizing microorganisms from managing soil phosphorus deficiency to mediating biogeochemical P cycle. Biology.

[94] Li H.-P., Han Q.-Q., Liu Q.-M., Gan Y.-N., Rensing C., Rivera W.L., Zhao Q., Zhang J.-L. (2023). Roles of phosphate-solubilizing bacteria in mediating soil legacy phosphorus availability. Microbiol. Res..

[95] Cheng Y., Narayanan M., Shi X., Chen X., Li Z., Ma Y. (2023). Phosphate-solubilizing bacteria: Their agroecological function and optimistic application for enhancing agro-productivity. Sci. Total Environ..

[96] Saeid A., Prochownik E., Dobrowolska-Iwanek J. (2018). Phosphorus solubilization by Bacillus species. Molecules.

[97] Ahmad I., Ahmad M., Hussain A., Jamil M. (2021). Integrated use of phosphate-solubilizing Bacillus subtilis strain IA6 and zinc-solubilizing Bacillus sp. strain IA16: A promising approach for improving cotton growth. Folia Microbiol..

[98] James N., Umesh M., Sarojini S., Shanmugam S., Nasif O., Alharbi S.A., Lan Chi N.T., Brindhadevi K. (2023). Unravelling the potential plant growth activity of halotolerant *Bacillus licheniformis* NJ04 isolated from soil and its possible use as a green bioinoculant on *Solanum lycopersicum* L.. Environ. Res..

[99] Gomez-Ramirez L.F., Uribe-Velez D. (2021). Phosphorus solubilizing and mineralizing *Bacillus* spp. contribute to rice growth promotion using soil amended with rice straw. Curr. Microbiol..

[100] Lili W., Shijun Z., Rui D., Jinbo Z., Shuquan J. (2022). Effects of bio-organic fertilizer and microbial agent on the growth, yield and quality of tomato. Soils Crops.

[101] Liu Y., Shi J., Feng Y., Yang X., Li X., Shen Q. (2013). Tobacco bacterial wilt can be biologically controlled by the application of antagonistic strains in combination with organic fertilizer. Biol. Fertil. Soils.

[102] Tripathi A., Meena B., Pandey K., Singh J. (2020). Microbial bioagents in agriculture: Current status and prospects. New Front. Stress Manag. Durable Agric..

[103] Lv L., Huang H., Lv J., Xu X., Cao D., Rao Z., Geng F., Kang Y. (2024). Unique dissolved organic matter molecules and microbial communities in rhizosphere of three typical crop soils and their significant associations based on FT-ICR-MS and high-throughput sequencing analysis. Sci. Total Environ..

[104] del Barrio-Duque A., Samad A., Nybroe O., Antonielli L., Sessitsch A., Compant S. (2020). Interaction between endophytic Proteobacteria strains and Serendipita indica enhances biocontrol activity against fungal pathogens. Plant Soil.

[105] Bovio-Winkler P., Guerrero L.D., Erijman L., Oyarzúa P., Suárez-Ojeda M.E., Cabezas A., Etchebehere C. (2023). Genome-centric metagenomic insights into the role of Chloroflexi in anammox, activated sludge and methanogenic reactors. BMC Microbiol..

[106] Yang W., Gong T., Wang J., Li G., Liu Y., Zhen J., Ning M., Yue D., Du Z., Chen G. (2020). Effects of compound microbial fertilizer on soil characteristics and yield of wheat (*Triticum aestivum* L.). J. Soil Sci. Plant Nutr..

[107] Javed Z., Tripathi G.D., Mishra M., Dashora K. (2021). Actinomycetes–the microbial machinery for the organic-cycling, plant growth, and sustainable soil health. Biocatal. Agric. Biotechnol..

[108] Chen Y., Li X., Li S., Xu Y. (2021). Novel-integrated process for production of bio-organic fertilizer via swine manure composting. Environ. Eng. Res..

