# Supplementary material for: Bacillus licheniformisYB06: A Rhizosphere–Genome-Wide Analysis and Plant Growth-Promoting Analysis of a Plant Growth-Promoting Rhizobacterium Isolated from Codonopsis pilosula
Source: Microorganisms. 2024 Sep 8;12(9):1861. doi: 10.3390/microorganisms12091861 (PMC11433706; doi:10.3390/microorganisms12091861)
Supplement: Supplementary file 1 [file microorganisms-12-01861-s001.zip › microorganisms-3193537-supplementary.pdf]

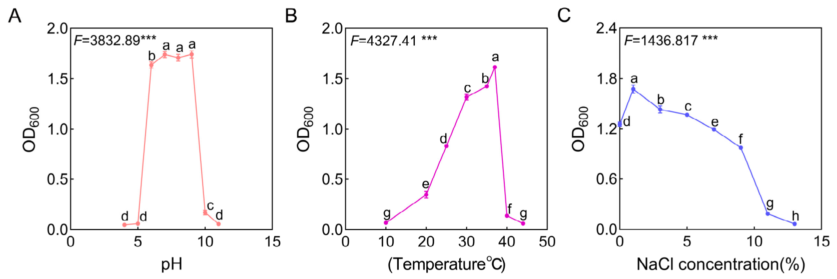

Figure S1. Influence of various factors on the growth of B. licheniformis YB06: (A) pH, (B) temperature, (C) NaCl concentration.

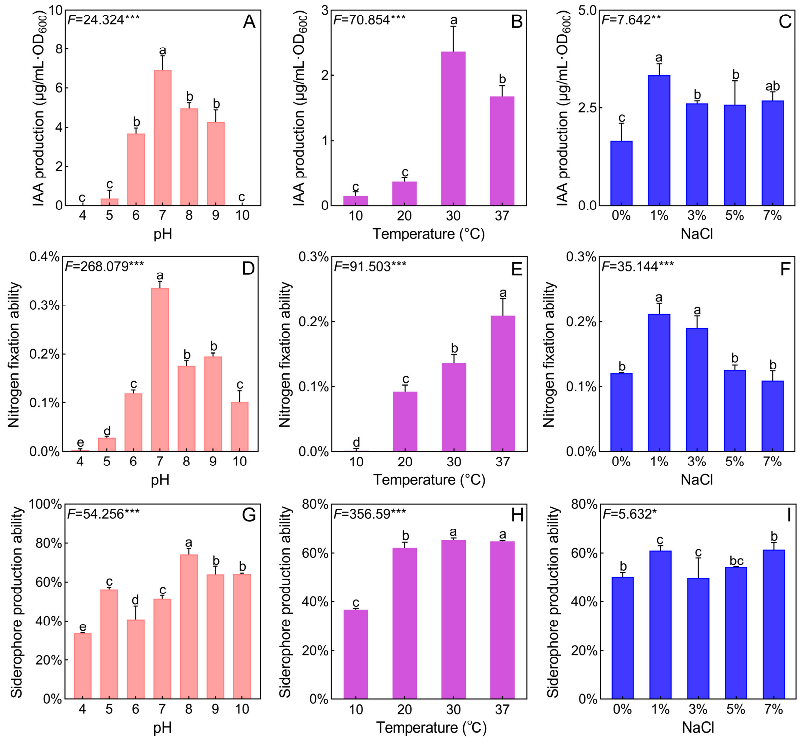

Figure S2. Effect of pH (A), temperature (B), and NaCl concentration (C) on indole-3-acetic acid (IAA) production by the strain. Effect of pH (D), temperature (E), and NaCl concentration (F) on nitrogen fixation capacity of the strain. Effect of pH (G), temperature (H), and NaCl concentration (I) on siderophore production by the strain.

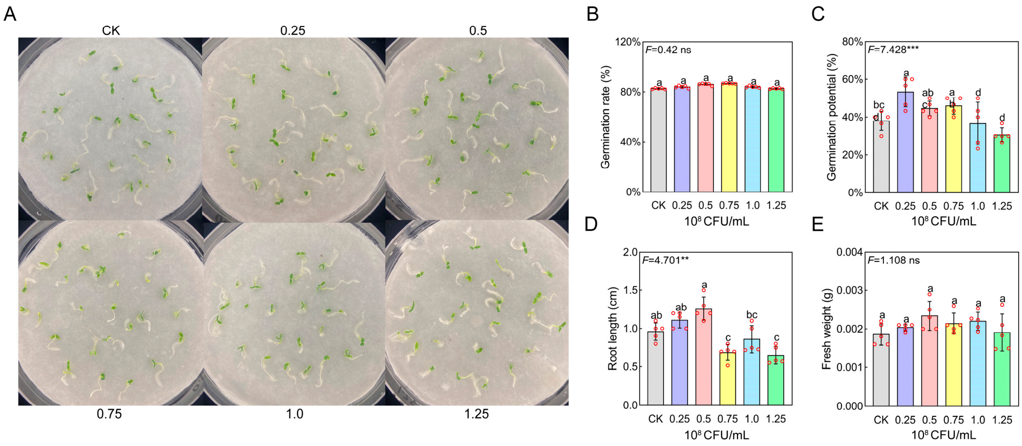

Figure S3. Effects of varying concentrations of *B. licheniformis* YB06 (10^8 CFU/mL) on the (A) Germination status of *C. pilosula* seeds after 14 days of cultivation (50 seeds per treatment, and ungerminated seeds were removed), (B) Germination rate of seeds after 14 days of cultivation, and (C) Germination potential of seeds after 14 days of cultivation. Effects of varying concentrations of *B. licheniformis* YB06 on the (D) Root length and (E) Fresh weight of C. *pilosula* seedlings. This was a preliminary experiment with one treatment group per concentration.

Table S1. Summary statistics of non-coding RNA prediction results.

Type	Number	Average length (bp)	Total Length (bp)	Percentage of total gene length (%)
tRNA	81	77	6253	0.15
16S rRNA	8	1550	12404	0.29
5S rRNA	8	116	928	0.02
23S rRNA	8	2930	23443	0.55
sRNA	31	101	3145	0.07

Table S2. Statistics of gene island prediction results.

ID	Start	End	Length (bp)	Number of coding genes
GI_1	935271	1022772	87502	103
GI_2	1417660	1424713	7054	7
GI_3	2869178	2881634	12457	14
GI_4	3219641	3226199	6559	8
GI_5	3436946	3471325	34380	43
GI_6	3477330	3488112	10783	17

Note: Genomic island GI_1, spanning 87,502 base pairs (bp) from positions 935,271 to 1,022,772, encompasses 103 coding genes (NR984_04630 - NR984_05245). GI_2, located at positions 1,417,660 to

1,424,713, spans 7,054 bp and contains seven predicted coding genes (NR984_07285 - NR984_07315). GI_3, spanning 12,457 bp from positions 2,869,178 to 2,881,634, encompasses 14 predicted coding genes (NR984_14925 - NR984_15005). GI_4 and GI_6, located at positions 3,219,641-3,226,199 and 3,477,330-3,488,112, respectively, span 6,559 bp and 10,783 bp, and contain eight (NR984_16815 - NR984_16845) and 17 (NR984_18170 - NR984_18255) predicted coding genes, respectively. GI_5, spanning 34,380 bp from positions 3,436,946 to 3,471,325, encompasses 43 predicted coding genes (NR984_17905 - NR984_18130).

GI_1 predominantly encodes proteins involved in nucleotide metabolism and DNA recombination, including a nucleotidyltransferase-like protein (NR984_04640), a tyrosine recombinase (NR984_04735), an ATP-binding protein (NR984_04805), a glycosyltransferase (NR984_05180), a creatine kinase (NR984_04960), and an FAD-dependent thymidylate synthase (NR984_04945). GI_2 appears to be associated with bacteriophage infection, containing a phage portal protein (NR984_07285), a hypothetical protein (NR984_07290), and a protein with a transglycosylase SLT domain (NR984_07315). GI_3 primarily encodes proteins involved in signaling and regulation, notably a Phr family-secreted Rap phosphatase inhibitor (NR984_14985) and a metallophosphoesterase (NR984_15005). GI_4 encompasses genes linked to amino acid metabolism and redox reactions, including a histidine kinase (NR984_16820), a polyprenyl synthetase family protein (NR984_16830), and an SDR family oxidoreductase (NR984_16845). GI_5 harbors genes encoding proteases involved in protein degradation and turnover, specifically a peptidase G (NR984_18010) and a Clp protease (NR984_18070). GI_6 primarily encodes proteins implicated in nucleic acid and small molecule metabolism, such as a ribonuclease R (NR984_18245) and a carboxylesterase (NR984_18250).

Table S3. Summary of predicted prophages.

ID	Begin	End	Length (bp)	Gene number	Gene ID
Prophage_001	985193	1029681	127367	44	NR984_05050~NR984_05270
Prophage_002	1395191	1433551		49	NR984_07135~NR984_07375
Prophage_003	3445437	3489953		59	NR984_17980~NR984_18275

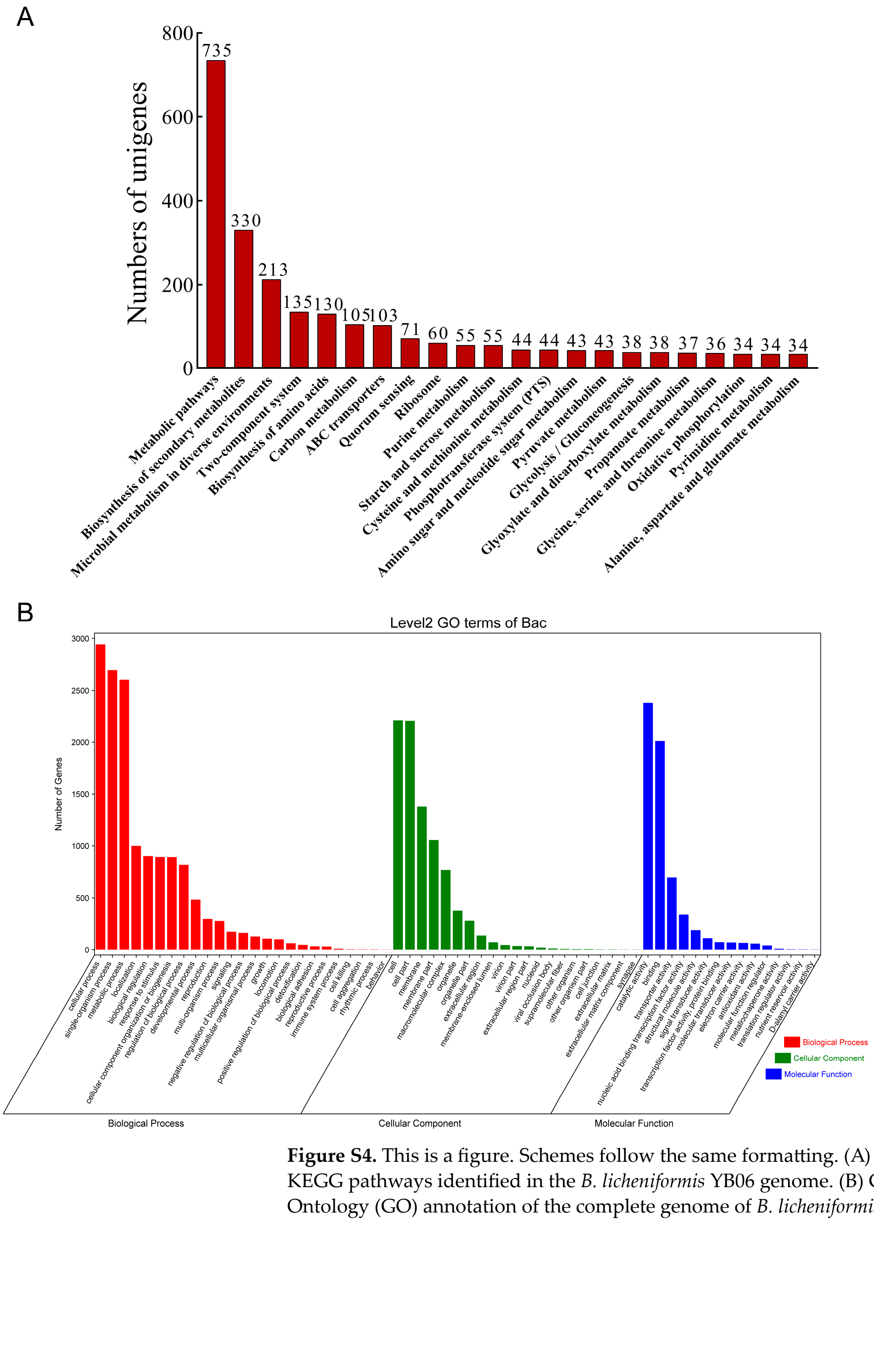

Figure S4. This is a figure. Schemes follow the same formatting. (A) Top 20 KEGG pathways identified in the *B. licheniformis* YB06 genome. (B) Gene Ontology (GO) annotation of the complete genome of *B. licheniformis* YB06.

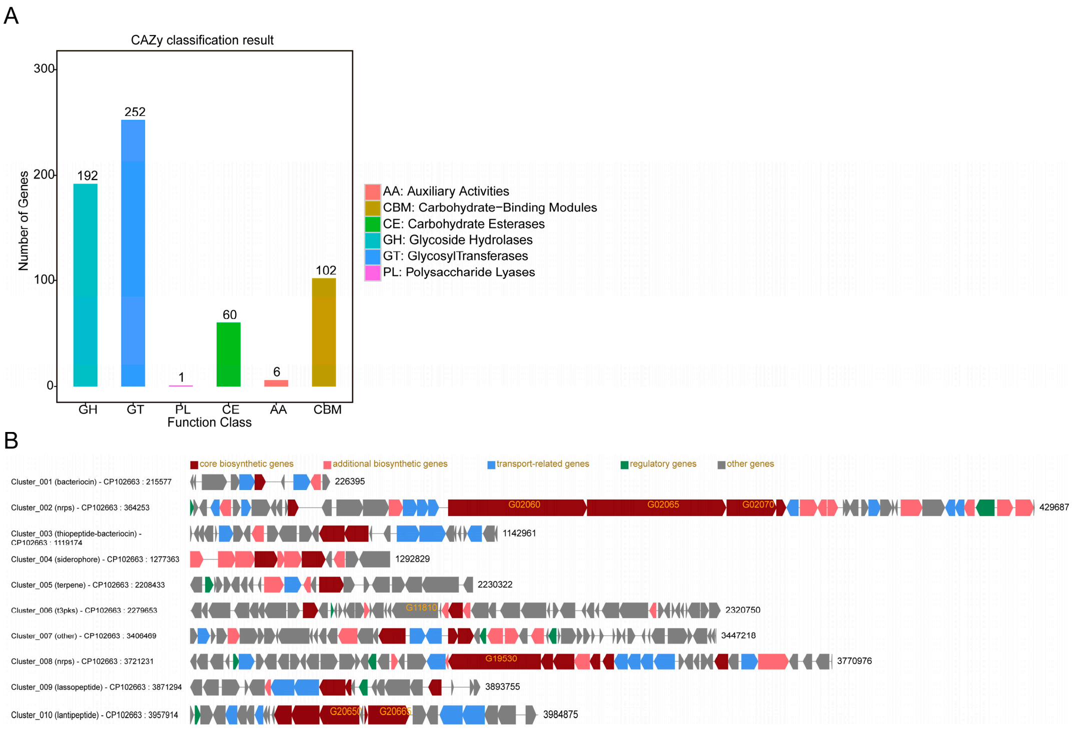

Figure S5. (A) Classification of carbohydrate-active enzymes (CAZymes) identified in the genome of *B. licheniformis* YB06. (B) Predicted secondary metabolite gene clusters in the genome of *B. licheniformis* YB06.

Table S4. Summary of identified virulence factors.

VFDB ID	Identity	Description
VFG000079	78.5	*clpC*
VFG049197	85.93	*clpB*
VFG010484	83.7	*htpB*
VFG000077	80.71	*clpP*
VFG020563	90.91	*invA*

Table S5. Summary of identified virulence factors.

ARO	Model_type	Drug Class	Resistance Mechanism	AMR Gene Family	ORF_ID
3003285	protein variant model	rifamycin antibiotic	antibiotic target alteration; antibiotic target replacement	rifamycin-resistant beta-subunit of RNA polymerase (rpoB)	NR984_00700
3003807	protein overexpression model	fluoroquinolone antibiotic; cephalosporin; glycylcycline;	antibiotic target alteration;	resistance-nodulation-cell division (RND)	NR984_05690 NR984_01825 NR984_02875 NR984_03235

		penam; tetracycline antibiotic; rifamycin antibiotic; phenicol antibiotic; triclosan	antibiotic efflux	antibiotic efflux pump	NR984_06295 NR984_09145 NR984_09955 NR984_10155 NR984_10160 NR984_11300 NR984_15615 NR984_17190 NR984_17445 NR984_18275
3004562	protein variant model	fluoroquinolone antibiotic	antibiotic target alteration	fluoroquinolone resistant gyrB	NR984_00030 NR984_10355 NR984_14490
3003479	protein overexpression model	tetracycline antibiotic	antibiotic target alteration; antibiotic efflux	major facilitator superfamily (MFS) antibiotic efflux pump	NR984_01810 NR984_02190 NR984_04315 NR984_04610 NR984_07715 NR984_07730 NR984_08360 NR984_10100 NR984_10205 NR984_11035 NR984_11490 NR984_11930 NR984_15035 NR984_18490
3003381	protein overexpression model	fluoroquinolone antibiotic; cephalosporin; glycylcycline; penam; tetracycline antibiotic; rifamycin antibiotic; phenicol antibiotic; triclosan	antibiotic target alteration; antibiotic efflux	ATP-binding cassette (ABC) antibiotic efflux pump; major facilitator superfamily (MFS) antibiotic efflux pump; resistance-nodulation-cell division (RND) antibiotic efflux pump	NR984_01405 NR984_01585 NR984_01880 NR984_03175 NR984_03985 NR984_04885 NR984_05700 NR984_07185 NR984_07755 NR984_10015 NR984_10545 NR984_13970 NR984_15165 NR984_21345

Note: A total of 329 genes were identified.

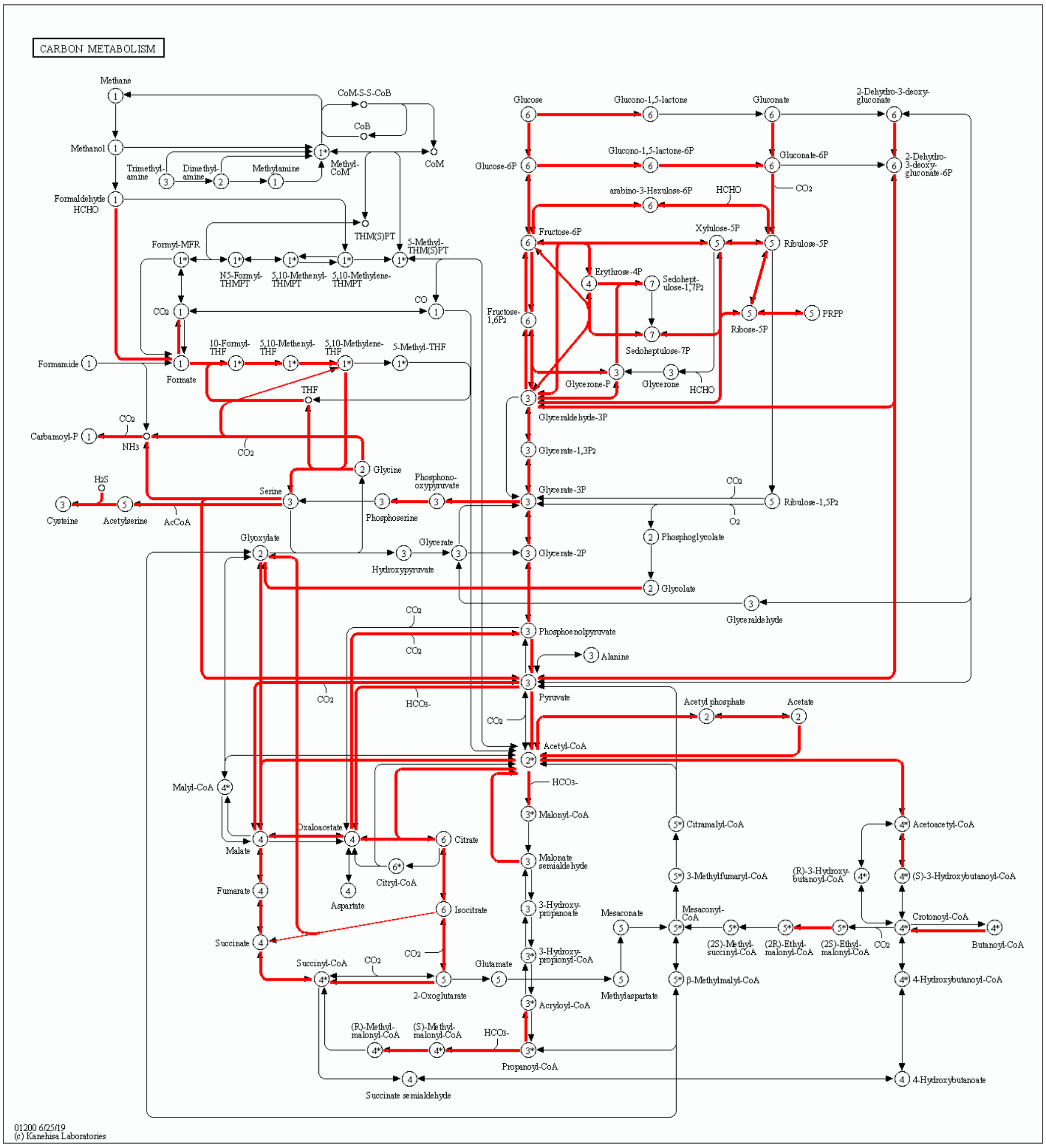

Figure S6. Carbon metabolism pathways in *B. licheniformis* YB06.

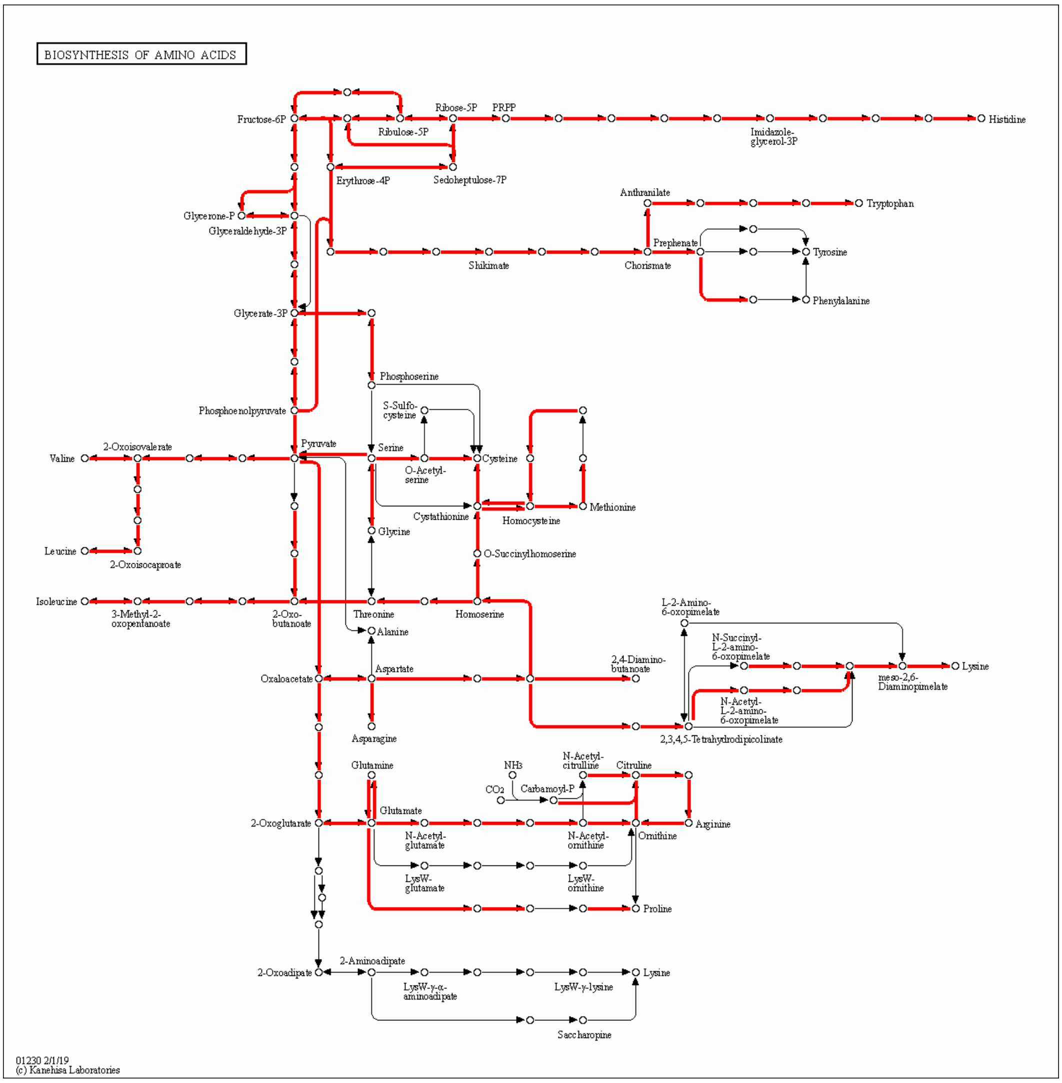

Figure S7. Amino acid metabolism pathways in *B. licheniformis* YB06.

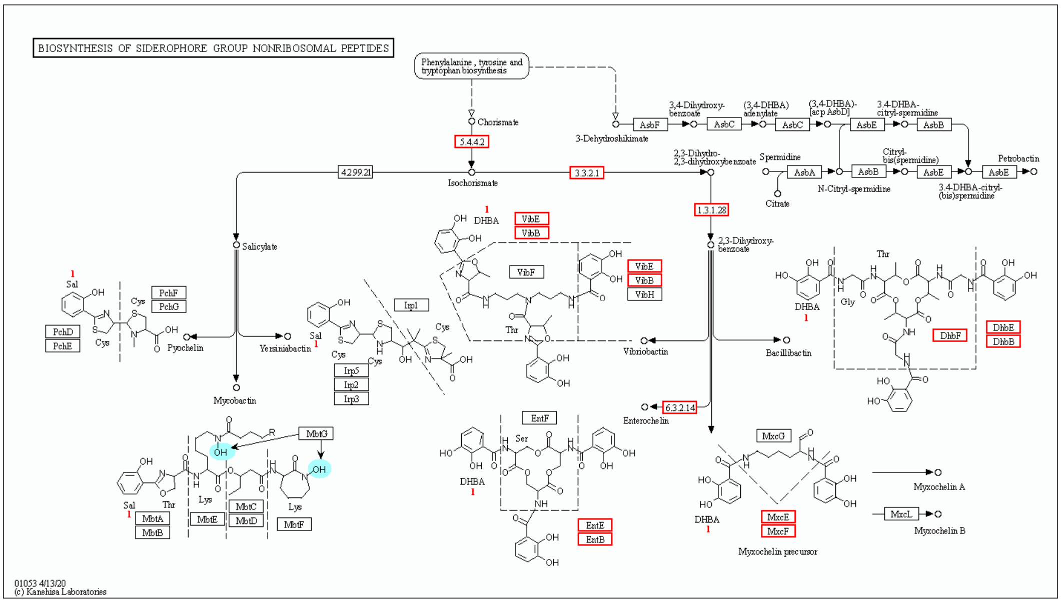

Figure S8. Biosynthesis of siderophore group nonribosomal peptides in *B. licheniformis* YB06.

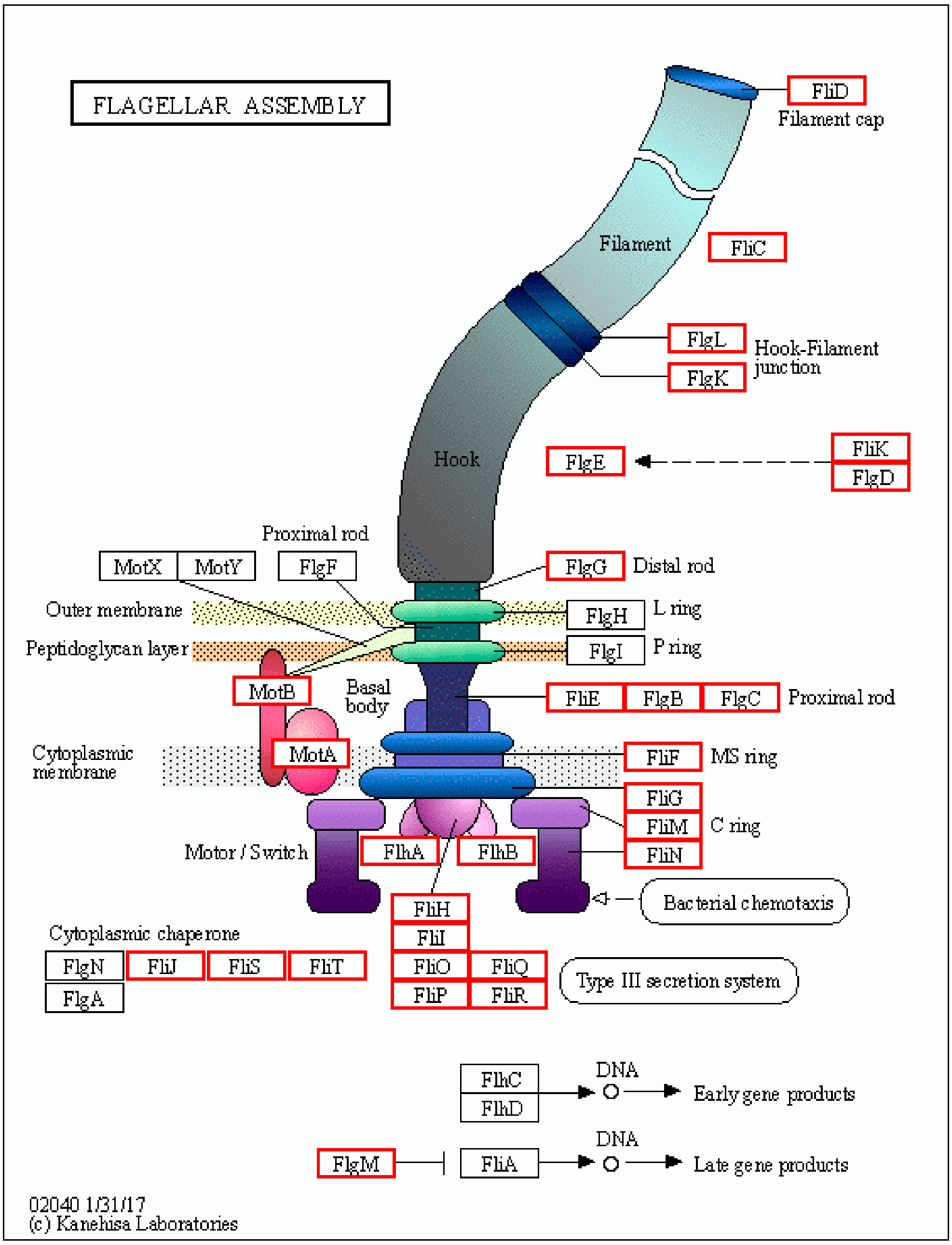

Figure S9. Flagellar assembly pathway in *B. licheniformis* YB06.

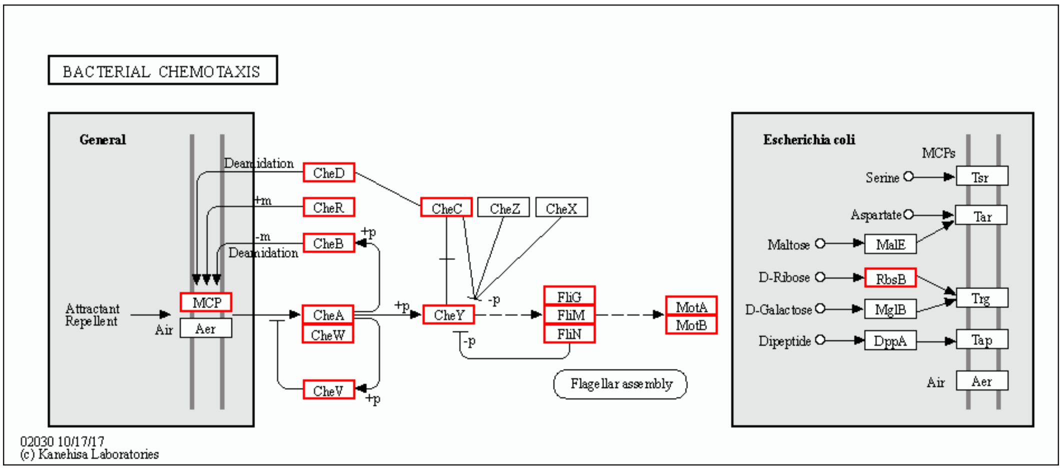

Figure S10. Chemotaxis-related proteins and gene pathways in *B. licheniformis* YB06.
